# Marine Phytoplankton Temperature versus Growth Responses from Polar to Tropical Waters – Outcome of a Scientific Community-Wide Study

**DOI:** 10.1371/journal.pone.0063091

**Published:** 2013-05-21

**Authors:** Philip W. Boyd, Tatiana A. Rynearson, Evelyn A. Armstrong, Feixue Fu, Kendra Hayashi, Zhangxi Hu, David A. Hutchins, Raphael M. Kudela, Elena Litchman, Margaret R. Mulholland, Uta Passow, Robert F. Strzepek, Kerry A. Whittaker, Elizabeth Yu, Mridul K. Thomas

**Affiliations:** 1 NIWA Centre for Chemical and Physical Oceanography, Department of Chemistry, University of Otago, Dunedin, New Zealand; 2 National Institute of Water and Atmosphere, Greta Point, Wellington, New Zealand; 3 Graduate School of Oceanography, University of Rhode Island, Narragansett, Rhode Island, United States of America; 4 Department of Biology, University of Southern California, Los Angeles, California, United States of America; 5 University of California Santa Cruz, Santa Cruz, California, United States of America; 6 Department of Ocean, Earth and Atmospheric Sciences, Old Dominion University, Norfolk, Virginia, United States of America; 7 Michigan State University, Kellogg Biological Station, Hickory Corners, Michigan, United States of America; 8 Department of Life Sciences, University of California Santa Barbara, Santa Barbara, California, United States of America; 9 Department of Chemistry, University of Otago, Dunedin, New Zealand; Institute of Marine Research, Norway

## Abstract

“*It takes a village to finish (marine) science these days*”

*Paraphrased from Curtis Huttenhower (the Human Microbiome project)*

The rapidity and complexity of climate change and its potential effects on ocean biota are challenging how ocean scientists conduct research. One way in which we can begin to better tackle these challenges is to conduct community-wide scientific studies. This study provides physiological datasets fundamental to understanding functional responses of phytoplankton growth rates to temperature. While physiological experiments are not new, our experiments were conducted in many laboratories using agreed upon protocols and 25 strains of eukaryotic and prokaryotic phytoplankton isolated across a wide range of marine environments from polar to tropical, and from nearshore waters to the open ocean. This community-wide approach provides both comprehensive and internally consistent datasets produced over considerably shorter time scales than conventional individual and often uncoordinated lab efforts. Such datasets can be used to parameterise global ocean model projections of environmental change and to provide initial insights into the magnitude of regional biogeographic change in ocean biota in the coming decades. Here, we compare our datasets with a compilation of literature data on phytoplankton growth responses to temperature. A comparison with prior published data suggests that the optimal temperatures of individual species and, to a lesser degree, thermal niches were similar across studies. However, a comparison of the maximum growth rate across studies revealed significant departures between this and previously collected datasets, which may be due to differences in the cultured isolates, temporal changes in the clonal isolates in cultures, and/or differences in culture conditions. Such methodological differences mean that using particular trait measurements from the prior literature might introduce unknown errors and bias into modelling projections. Using our community-wide approach we can reduce such protocol-driven variability in culture studies, and can begin to address more complex issues such as the effect of multiple environmental drivers on ocean biota.

## Introduction

To date, much of the progress in understanding how climate change will manifest itself in the ocean has come from projections obtained from global modelling experiments using general circulation or coupled ocean atmosphere models [1], [2]. These types of models provide predictions of how bulk properties such as phytoplankton stocks (based on the proxy chlorophyll *a*) or ecosystem-level properties, such as downward export flux, will be altered by climate in the coming decades. However, at present many environmental projections from models – for example global maps of altered upper ocean temperature or nutrient concentrations - cannot be put into the urgently-needed wider context of the biological or ecological implications resulting from climate change. Such under-utilisation of model outputs is due to the current dearth of information on the physiological performance (often expressed as fitness versus environment, [3]) of many phytoplankton groups, species or ecotypes that are key players in the biogeochemical cycling of major (C, N, P) and minor (Fe, Zn, Co) elements in the ocean [4]. Given that phytoplankton photosynthesis and nitrogen fixation make major contributions to global C [5], [6] and N [7], [8] inventories, respectively, they can potentially drive significant feedbacks on climate change. However, the sign and magnitude of most biologically-mediated feedbacks are not yet known [9]. Both global modelling experiments [10] and time-series data [11] reveal that the upper ocean is already warming in many regions (excluding upwelling regions, [12] and is highly likely to continue warming as a result of a changing climate.

The temperature of the upper ocean is a fundamental control on phytoplankton metabolic processes [13], [14] and sets the biogeographical boundaries or biomes of major phytoplankton groups [15], [16]. As such, temperature response functions of phytoplankton are included in several widely used productivity models [17], leading to sensitivity in global primary production models to projected warmer surface ocean temperatures [6]. Furthermore, experiments in which multiple environmental properties are manipulated reveal that temperature has significant interactive (i.e. synergistic or antagonistic) effects with other properties such as carbon dioxide and/or iron concentrations, and on phytoplankton processes such as growth, photo-physiology, and calcification [18], [19], [20], [21]. There have been several syntheses of temperature versus growth rate relationships for a range of laboratory-cultured phytoplankton [22] to look at generic relationships across a wide range of ocean temperatures. However, most comparative studies examining the influence of temperature on phytoplankton physiology were conducted several decades ago [23], [24] and so such syntheses have had to rely upon available datasets that were collected using a range of protocols, both for culturing and estimation of growth rates. Moreover, many of the lab-cultured isolates used in these studies were isolated from the coastal ocean and/or were long-institutionalised (i.e. decades), and easily cultured “weed” species [22].

At present, there is a growing disjoint between the proliferation, improved accuracy and resolution of model projections of how oceanic conditions will change in the next 4–5 decades [25], and the availability of physiological datasets needed to contextualise such environmental projections. For example, the availability of datasets describing both the temperature optima for phytoplankton growth and the thermal limits (termed thermal niche width) at which pronounced decreases in physiological performance occurs, is limited, particularly for open ocean phytoplankton groups that drive major biogeochemical cycles [26]. Moreover, because different experimental approaches have been used to conduct laboratory culture experiments and calculate growth rates, the validity of comparing many of the datasets is questionable. Obtaining datasets that reveal the fundamental responses of organisms to altered ocean conditions, such as temperature, that are well-replicated across relevant physiological or environmental ranges for specific organisms is time-consuming, and funding may be difficult to obtain due to perceptions that such research has already been conducted in previous decades [22] or is unnecessarily simplistic.

However, these datasets are fundamental for parameterising models to make informed projections of how oceanic biota and ecosystems will respond to change in the many biomes that make up the global ocean [15]. Given that global change is occurring, and is highly likely to continue do so in the coming decades, we cannot afford to delay obtaining and employing such datasets to advise our models [27].

A recent development in other major disciplines, faced with similarly complex systems, has been the adoption of a community-based approach to tackle the issues associated with a daunting number of permutations. In the disciplines of astronomy [28] and biochemistry/protein-folding [29] unprecedented rapid progress has been made through implementation and fostering of such community-wide initiatives. We maintain that in order to address issues of complex system science – e.g., multiple oceanic biomes with many different phytoplankton species, and a wide range of potential algal responses to environmental change – such community-wide efforts are necessary. However, because sufficient data relating to phytoplankton responses to climate change variables do not exist we carried out a pilot community-wide laboratory study. As part of this study we established a common laboratory approach for conducting experiments and then using this approach, we measured growth responses of cultured representatives from key phytoplankton groups to temperature.

**Figure 1:**
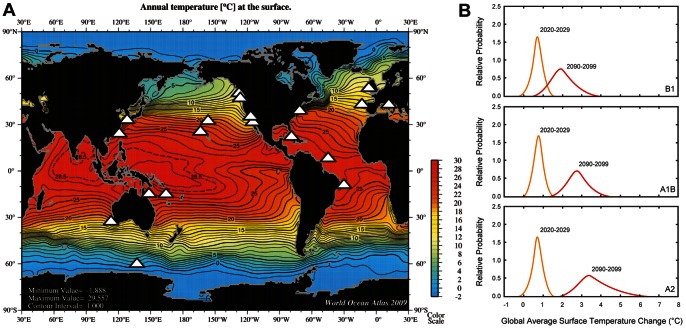
Summary of the locations at which the species/strains were initially isolated. A) Overlaid (locales denoted by white stars) on a global map of satellite sea surface temperature (°C, from World Ocean Atlas, [91]); B) Projected surface ocean temperature changes for the early and late 21st century relative to the period 1980–1999. The global average surface ocean temperature change is plotted against the relative probabilities of estimated global average warming from several different AOGCM and Earth System Model of Intermediate Complexity. The data are for average projections for the B1, A1B, and A2 SRES scenarios. Plot is from IPCC AR4 [104].

Cultured isolates were examined originating from polar to tropical oceanic regions, and from coastal to remote offshore waters (Table 1). The phytoplankton included eukaryotes such as a Southern Ocean diatom, isolated in waters of ∼3°C and used for iron and photo-physiology studies [30], [31] to prokaryotic nitrogen fixers (diazotrophs), isolated from tropical oligotrophic waters, being investigated for their response to “greenhouse” ocean conditions (i.e. a higher CO_2_ warmer ocean, Fu et al., unpublished data, Hutchins et al. unpublished data) (Figure 1a). Other nearshore non-diazotrophic cyanobacterial species also being examined for their response to future ocean conditions (higher temperature and pCO_2_; Ozmon et al., unpublished data), provided a contrast with offshore species. In addition, eukaryotic and mixotrophic dinoflagellate species involved in estuarine and coastal harmful algal blooms (HABs) and nearshore eutrophication were examined [32] as blooms of these groups of phytoplankton are thought to be favored under future climate scenarios [33]. A further contrast was provided between species in the diatom genus *Thalassiosira* that have been comprehensively studied physiologically and genetically, such as *Thalassiosira pseudonana* [34], [35], [36], *Thalassiosira weissflogii* [23], [37], [38] and *Thalassiosira rotula* [39], [40] to those about which relatively little is known – such as the small diazotroph *Crocosphaera watsonii* [41] and the polar diatom *Proboscia enermis* [42] that have only recently been isolated.

**Table 1:** The provenance, distribution and environmental relevance of each of species/strains used in this study.

Species/strains	Provenance	Environmental relevance	Regional distribution
*Akashiwo sanguinea* (4 strains)	Isolated (2006 to 2010) during Harmful Algal Bloom (HAB) events in NE Pacific BWA (NWFSC-605): 48.27°N, 124.68°W GBB: 47.90°N, 124.63°W RMB: 36.96°N, 122.01°W YRB: 33.84°N, 118.39°W	Dinoflagellate implicated in two large-scaleHAB events possibly due to changingenvironmental conditions [71], [73], [105]	Mid-latitude coastal waters, including the Black Sea
*Thalassiosira weissflogii*	CCMP1053, 39.50N 9.33W, isolated in 1973	Very cosmopolitan coastal species that grows under a wide variety of environmental conditions. Very well studied (physiology, environmental conditions), partial genetic sequence	Coastal Atlantic, Pacific, Asian waters
*Thalassiosira rotula* (6 strains)	JpnTR18: 34.17 N, 133.33 E, Isolated in 2007 CCMP3264, 40.49N 14.14E, isolated in 2008 CCAP1085_21, 40.956 N, 14.25 E, isolated in 2008 P17F4, 49.65N, 127.44W isolated in 2007 CMP3096, 49.65N 127.43W, isolated in 2007 CCMP1647, 40.95N, 14.25E, isolated in 1993	Cosmopolitan diatom in near-shore and some offshore regions that grows under a wide variety of environmental conditions and can form large blooms.	Temperate waters
*Thalassiosira pseudonana* (6 strains)	CCMP 1011, 17.79N, 64.82E CCMP 1012, 31.99S, 115.83W CCMP 1013 (53.28N, 3.83W) CCMP 1014 (28 N, 155E) CCMP 1015 (48.54N, 123.01E), CCMP 1335 (40.76N, 72.82E)	Cosmopolitan diatom in near-shore regions that grows under a wide variety of environmental conditions and can form large blooms.	Temperate waters
*Proboscia inermis*	Isolated in the Pacific sector of S. Ocean(16°S 145°E), austral summer 2002 (see [30])	Large diatom, bloom former	Southern Ocean polar
*Trichodesmium erythraeum*	Tricho RLI,1997 Tricho KO4, 2006 Tricho2175, 2007	Colonial N fixer	Great Barrier ReefS Pacific 15°03 S; 155°02 E W Equatorial Atlantic 7°32 N; 49°15W
Crocosphaera *watsonii*	Cro WH 3A, March 2002Cro WH84, March 2002 CroWH0005 March 2000	Unicellular N fixer (3–4.5 µm)	North Atlantic 6°58.78 N; 49°19.70 W South Atlantic 11°42.12 S; 32°00.64 W North Pacific 21°25.98 N; 157°47.29 W
Coastal *Synechococcus* (CCFWC 502) Cro WH84	West Florida Shelf and was obtained from Florida Wildlife Research Institute (FWRI) and maintained on f/2 medium [34] 4.5 µm, March 2002	Unicellular picophytoplankton	Atlantic 11°42.12 S; 32°00.64 W
*Prorocentrum donghaiense* CroWH0005	March 2000	Coastal dinoflagellate (4.3 µm)	Changjiang River estuary, coastal areas of Zhejiang province and Guangdong province and Hong Kong, Japan and South KoreaNorth Pacific 21°25.98 N; 157°47.29 W

In addition to testing the efficacy of this community-wide pilot study, this research provides insights as to how regional projections of warming might alter the physiological performance of phytoplankton groups/species that reside in distinctly different biomes across the world ocean (Figure 1). Our study also enables a comparison with a recent collation [43] of published data on phytoplankton temperature versus growth rate relationships that were made with a wide range of experimental protocols. It is critical to establish the value of such syntheses of the earlier literature, whether we can use their parameterizations describing physiological growth response of different phytoplankton to temperature, and assess if we can relate these parameterizations to modelled projections of primary production and biogeographic distributions in response to regional warming. Our dataset provides an example of what is required to better parameterise models and predict how phytoplankton (as well as other microbial and planktonic groups) will be affected by changing temperatures [44], [45], [46] and hence the degree to which pelagic ecosystems will be potentially restructured in the future.

## Results

### Temperature Growth Curves

**Figure 2:**
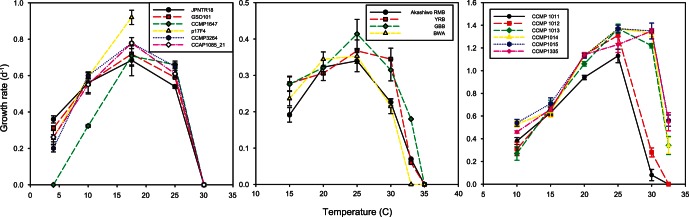
Thermal reaction norms for multiple strains of *Thalassiosira rotula* (left panel) *Akashiwo sanguinea* (central panel) and *Thalassiosira pseudonana* (right panel) used in our study.

**Figure 3:**
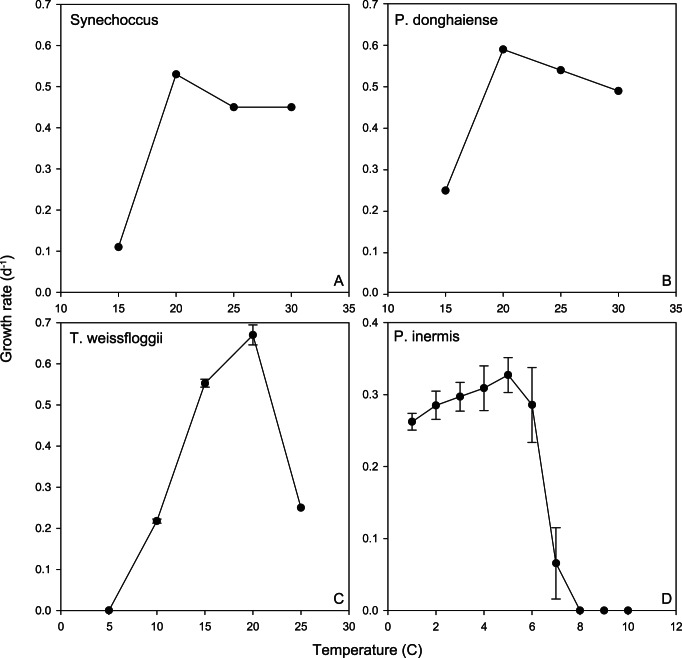
Thermal reaction norms for tropical to polar phytoplankton (single strains) used in our study.

**Figure 4:**
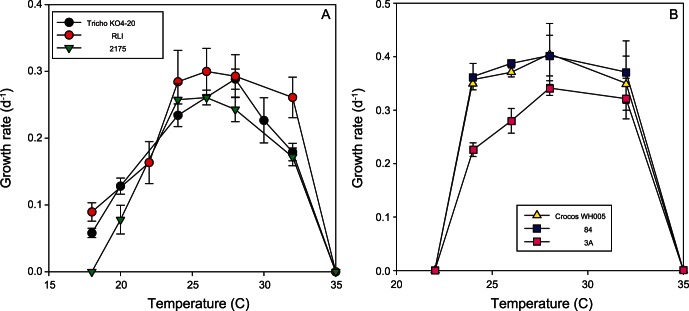
Thermal reaction norms for multiple strains of the tropical a) *Trichodesmium erythraeum;* b) *Crocosphaera watsonii* phytoplankton used in our study.

The growth curves (hereafter termed reaction norms) for each species or strain are presented for polar and temperate species in Figures 2 and 3, and for tropical species in Figure 4 (see also Figures S1 and S2). The maximum growth rates ranged from 0.3 to 1.4 d^−1^ for the polar and temperate species, and were ∼0.3 d^−1^ for all of the tropical nitrogen fixers (Figures 2, 3, and 4). The shapes of the reaction norms varied considerably from strongly asymmetric for the polar diatom to a more symmetric response for warmer water species and strains. Interspecific differences in the reaction norms were determined using species for which multiple strains were examined and a bootstrapping approach. The reaction norms of remaining species, where just a single strain was examined, were compared qualitatively. We first describe the main features of the reaction norms for all species, and then provide a statistical comparison of species with multiple strains.

The reaction norms of the diatoms species examined differed considerably. For the polar diatom (Figure 3D), a temperature increase of 3°C (relative to the isolation temperature ∼3°C, c.f. Figure 1B) resulted in a 25% increase in growth rate (µ d^−1^), but then a further 1°C of warming caused a rapid decrease in µ followed by mortality. All three temperate diatom species (Figures 2 and 3 C) had broadly similar shaped reaction norms, exhibiting up to a four-fold increase in µ as temperatures increased but no further increases in growth rates at temperatures >20°C, or 25°C in the case of *Thalassiosira rotula* (Figure 2).

For the tropical species, the unicellular nitrogen fixing isolates (*Crocosphaera watsonii*) had a similar reaction norm curve to the ubiquitous cyanobacterium *Synechococcus* (Figure 3A), a non-diazotrophic unicellular cyanobacteria, as did the three diazotrophic *Trichodesmium* isolates (Figure 4). Each diazotroph had a plateau in growth rate at 24 to 28°C. Growth rates dropped off at the upper end of this temperature range in a similar manner for both the unicellular and colonial nitrogen fixers (i.e. to zero by 35°C). However, compared to *Crocosphaera* the biggest difference between these reaction norms is that *Trichodesmium* has lower minimum temperature limits and lower maximum growth rates (Figure 4).

The dinoflagellate, *A. sanguinea*, displayed maximum growth rates at 25°C and an upper temperature limit of 30 to 33°C (Figure 2). Attempts to grow the strains at 35°C failed repeatedly with some strains remaining vegetative (but with no discernible growth) followed by mortality. All strains had robust growth rates at 15°C. Attempts to grow the strains at 10°C resulted in little/no growth but with all strains maintaining vegetative populations, indicating a lower temperature tolerance (for growth) of between 10–15°C and an ability to survive at relatively low temperatures compared to the thermal optimum. The dinoflagellate *P. donghaiense* exhibited a ∼3-fold increase in growth rate between 15 and 20°C, then exhibited ∼15% decreases in growth rate at 30°C (Figure 3B).

### Intraspecific Variations in Growth Rate

Multiple strains were examined for the dinoflagellate *A. sanguinea* and the diatoms *Thalassiosira rotula* and *Thalassiosira pseudonana* at temperatures ranging from 4°C to 35°C. The coefficient of variation (CV) was examined to assess variability within species at each temperature. For *A. sanguinea*, the CV ranged from 8–82% over a temperature range of 15–33°C (Table 2). The CV for *Thalassiosira pseudonana* ranged from 8% to 133% across a range of 10 to 32.5°C. For *Thalassiosira rotula*, the CV had a range of 9–53% across a temperature range of 4–25°C (no growth at 30°C). In general, these species exhibited more variation in growth rates among strains at the low and high temperature extremes (Table 2). For example, in *A. sanguinea* the upper temperature boundary (33°C) had a CV that was an order of magnitude higher than the mid-range of the reaction norm (20°C). As noted above, strains also exhibited varying survival at the lowest and highest temperature ranges. Contrary to what one might expect, the least and greatest reduction in growth at elevated temperatures (33°C) occurred in the most northerly strains (BWA and GBB) (Figure 2 middle panel), both isolated from the state of Washington, while the most southerly strain (YRB) showed the least sensitivity to low temperature (15°C), suggesting that geographical location does not necessarily correlate with optimal conditions for these coastal strains. This highlights the importance of evaluating multiple strains for a given region, since substantially different results would be obtained for the two isolates from Washington if one of those strains were used as representative of the species.

**Table 2:** Number of strains measured, mean growth rate and coefficient of variation amongst strains for each species and temperature.

Species	Temperature	Number of strains	Mean growth rate(m day^−1^)	Coefficient ofVariation (%)	ANOVAp value
*Akashiwo sanguinea*	15	4	0.249±0.044	17.7	<0.001
*Akashiwo sanguinea*	20	4	0.329±0.026	7.9	**0.272**
*Akashiwo sanguinea*	25	4	0.391±0.053	13.6	<0.001
*Akashiwo sanguinea*	30	4	0.262±0.067	25.6	<0.001
*Akashiwo sanguinea*	33	4	0.092±0.075	81.5	<0.001
*Crocosphaera watsonii*	22	3	No growth		
*Crocosphaera watsonii*	24	3	0.304±0.060	19.7	<0.001
*Crocosphaera watsonii*	26	3	0.414±0.100	24.2	<0.001
*Crocosphaera watsonii*	28	3	0.458±0.088	19.2	<0.001
*Crocosphaera watsonii*	32	3	0.408±0.070	17.2	0.001
*Crocosphaera watsonii*	35	3	No growth		
*Thalassiosira pseudonana*	10	6	0.412±0.139	33.7	<0.001
*Thalassiosira pseudonana*	15	6	0.662±0.103	15.6	**0.803**
*Thalassiosira pseudonana*	20	6	1.090±0.082	7.5	<0.001
*Thalassiosira pseudonana*	25	6	1.290±0.154	11.9	0.025
*Thalassiosira pseudonana*	30	6	0.934±0.556	59.5	<0.001
*Thalassiosira pseudonana*	32.5	6	0.236±0.313	132.6	<0.001
*Thalassiosira rotula*	4	6	0.227±0.120	52.9	<0.001
*Thalassiosira rotula*	10	6	0.531±0.116	21.8	0.005
*Thalassiosira rotula*	17.5	6	0.759±0.128	16.9	0.031
*Thalassiosira rotula*	25	5	0.611±0.056	9.2	0.021
*Thalassiosira rotula*	30	5	No growth		
*Trichodesmium erythraeum*	16	3	No growth		
*Trichodesmium erythraeum*	18	3	0.064±0.021	32.8	<0.001
*Trichodesmium erythraeum*	20	3	0.120±0.019	15.8	0.001
*Trichodesmium erythraeum*	22	3	0.162±0.027	16.7	0.013
*Trichodesmium erythraeum*	24	3	0.264±0.020	7.6	**0.071**
*Trichodesmium erythraeum*	26	2	0.279±0.026	9.3	0.029
*Trichodesmium erythraeum*	28	3	0.275±0.027	9.8	0.004
*Trichodesmium erythraeum*	32	3	0.194±0.040	20.6	0.004
*Trichodesmium erythraeum*	35	3	No growth		
					

Analysis of variance was used to test for intraspecific differences in growth rates at each temperature examined (α = 0.05). Temperatures at which intraspecific variation was not significant are listed in bold.

The N_2_ fixers, *C. watsonii* and *T. erythraeum* generally had lower ranges of CV’s across their temperature range (17–24% and 8–33%, respectively) than for the temperate diatoms. With few exceptions, there were significant differences in growth rates among strains within a species (p<0.05, Table 2). At every temperature tested, there were significant intraspecific differences in µ amongst *Thalassiosira rotula* strains and *C. watsonii* strains. For *Thalassiosira pseudonana*, *A. sanguinea* and *T. erythraeum*, significant differences in growth rate occurred at every temperature except 15, 20 and 24°C, respectively (Table 2).

The reaction norm curves for each species provided the opportunity to examine growth response over a range of temperatures. For example, each of the strains of the cosmopolitan diatom species *Thalassiosira rotula* (Table 1) was characterised by similar reaction norms, except for an isolate from the Mediterranean (CCMP 1647, isolated in 1993 as opposed to post 2007 for the other strains), which survived under a narrower range of temperatures than the others. Other strains isolated from the Mediterranean (CCAP 1085_21, CCMP 3264) had broader reaction norms. Strains of the temperate dinoflagellate *A. sanguinea* **(**isolated from the N Pacific, Table 1) exhibited strikingly different reaction norms with respect to temperature. For example strain YRB continued to grow at rates close to µ_max_ at temperatures >25°C, whereas strains BWA and GBB had a pronounced decrease in µ above 26°C. In the case of *Thalassiosira pseudonana*, the six strains appear to split into two groups in their temperature responses. Strains CCMP 1011 and 1012 have lower optima and narrower niches than CCMP 1013, 1014, 1015 and 1335 (Figure 2, right panel). However, these groupings appear to be unrelated to the different locales that they were isolated from.

### Interspecific Variation in Thermal Traits

The five species for which multiple strains were tested (*A. sanguinea, C. watsonii, Thalassiosira pseudonana, Thalassiosira rotula, and Trichodesmium erythraeum*) differed significantly in their trait distributions (Table 3). The distribution of delta AICc values enabled us to evaluate whether interspecific differences in thermal traits existed, and permitted us to incorporate our uncertainty in the model fits. For both optimal temperature and maximum growth rate, the 95% confidence intervals on the delta AICc were >0 indicating significant differences among species for these traits (Table 4). The confidence intervals for temperature niche width overlapped with zero, indicating that distributions of niche width cannot be easily distinguished based on species identity. However, this may be because our uncertainty in this trait is large (as estimated from the distribution of this trait in the bootstraps, Table 3), particularly in the case of *T. pseudonana*, *T. rotula* and *A. sanguinea*.

**Table 3:** Statistical comparison of the bootstrapping results for each of the three thermal traits Temperature optima, Maximum growth rate and temperate niche width (w).

Species	Strain	Temp. opt.upper. CI	Temp. opt.lower.CI	Max.growth.upper. CI	Max. growth.lower. CI	w. upper.CI	w. lowerCI
*T. erythraeum*	KO4_20	27.69	26.73	0.28	0.26	19.32	17.79
	GBRTRLI101	29.57	27.72	0.34	0.30	34.18	18.35
	21_75	27.61	26.13	0.28	0.25	17.45	16.48
*C. watsonii*	WH005	29.84	26.48	0.51	0.40	16.40	12.65
	WH84	30.02	26.35	0.52	0.40	16.34	12.69
	3A	30.17	28.43	0.41	0.35	14.35	12.87
*P. inermis*		4.32	2.97	0.35	0.30	59.39	1.16
*A. sanguinea*	RMB	22.72	21.46	0.36	0.33	24.11	22.30
	YRB	26.54	24.81	0.40	0.38	99.50	37.51
	GBB	25.86	23.09	0.44	0.39	63.95	24.58
	BWA	23.01	21.16	0.37	0.33	28.07	23.16
*P. donghaiense*		28.45	27.26	0.70	0.63	29.02	24.49
*T. pseudonana*	CCMP1011	23.98	19.15	1.15	0.86	47.24	22.52
	CCMP1012	24.23	20.64	1.30	1.06	27.99	22.45
	CCMP1013	26.97	25.99	1.52	1.40	62.20	27.91
	CCMP1014	27.16	25.96	1.63	1.41	103.30	42.20
	CCMP1015	27.12	26.17	1.59	1.40	93.39	46.09
	CCMP1335	27.44	25.89	1.57	1.29	87.04	32.95
*T. rotula*	JPNTR18	19.10	18.21	0.71	0.69	50.76	37.04
	CCMP3096	19.67	18.66	0.76	0.73	41.49	33.09
	CCMP1647	21.27	21.07	0.78	0.77	26.05	25.84
	CCMP3264	19.92	17.89	0.86	0.79	32.24	28.35
	CCAP1085_21	19.22	19.04	0.80	0.79	31.85	31.31
*T. weissflogii*	CCMP1053	20.04	19.33	0.70	0.66	21.47	20.34
*Synechococcus*	CCFWC 502	36.46	31.88	0.78	0.70	48.70	29.17

CI denotes confidence interval.

**Table 4:** Boot-strapping results for the five species with multiple strains that we studies.

Trait	delta AICc lower CI	delta AICc upper CI
T. Optimum	33.14	45.93
Niche width	-4.93	15.96
Max growth rate	49.26	59.79

If the entire 95% confidence interval of AICc values exceeded zero, we concluded that species identity was a useful predictor and that species differed in the distribution of the trait.

### Intraspecific Variations in Thermal Traits – Comparison with the Literature

**Figure 5:**
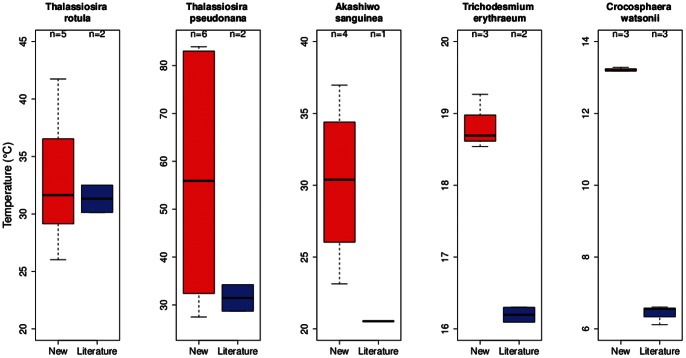
A comparison of the thermal trait, niche width (°C) using box and whisker plots, between previously published studies (using a wide range of experimental protocols, see [**43**] **) and the species/strains used in the present study.** The black bands denote the median value, the bottom and top of the red/blue boxes represent the 1st and 3rd quartile of the data respectively. The ‘whiskers’ extending from the boxes indicate the positions of the lowest & highest values in the data. If the sample size is small enough, the whiskers may not appear (e.g. if there are only 3 equally spaced points, the value represented as the 1st quartile is the lowest value).

**Figure 6:**
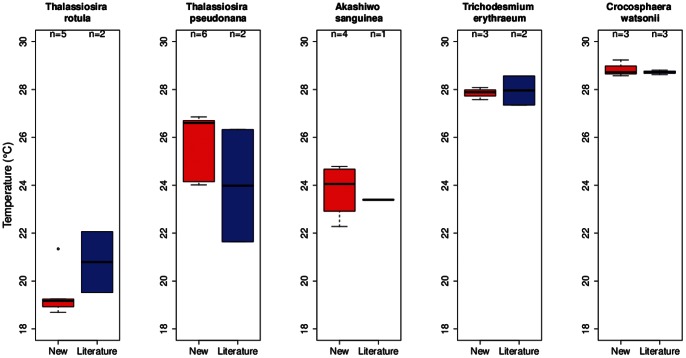
A comparison of the thermal trait, T_opt_ (°C) (box and whisker plots), between previously published studies (using a wide range of experimental protocols, see [43]) and the species/strains used in the present study. For details see Figure 5 caption.

**Figure 7:**
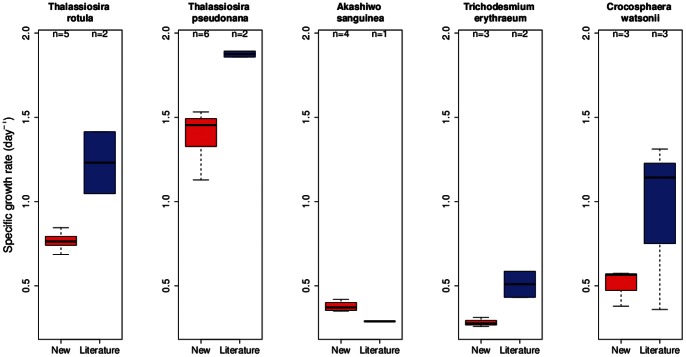
A comparison of the maximum specific growth rate (day ^−1^), using box and whisker plots, between previously published studies (using a wide range of experimental protocols, see [43]) and the species/strains used in the present study.

Of the eight species investigated in this study, we found previously published growth-temperature data for five common species (Table 5). For thermal niche width, there was less agreement between our study and prior published data (Figure 5). For example see *C. watsonii*, where the niche width is twofold larger from the prior literature relative to our study. In contrast, the thermal trait T_opt_ (Figure 6) was similar to previously published values for *T. erythraeum* and *Crochosphaera*, lower than previously reported for *Thalassiosira rotula*, and higher for *Thalassiosira pseudonana* and *A. sanguinea*. A comparison of maximum growth rates between the present and prior studies revealed significant differences for four of the five common species (Figure 7). In most cases, we investigated more strains than investigators did in the prior literature and hence we see greater variability in each of these thermal traits in the present study (Figures 5, 6, and 7; Figures S3, S4, S5, and S6).

**Table 5:** Summary of the environmental conditions used to culture phytoplankton species and strains in the present study.

		Protocol							
Organism	Laboratory	A.	B.	C.	D.	E.	F.	G.	H.
*Trichodesmium* spp.,*Crocosphaera* spp.	Hutchins/Fu	Yes	Yes (6 replicates)	Yes	Yes (150)	Yes	Yes	Yes	No^15^
*Thalassiosira pseudonana*	Litchman	Yes	Yes (6 replicates)	Yes	Yes (100)	Yes^6^	No^8^	No^12^	No^15^
*Proboscia inermis*	Boyd/Strzepek	Yes	Yes (6 replicates)	Yes	Yes (90)	Yes	Yes^9^	No^12^	Yes
*Prorocentrum donghaiense; Synechococcus*	Mulholland	No^1^	Yes	Yes^4^	Yes (35;100)	Yes	No^10^	Yes^13^	Yes
*Akashiwo sanguinea*	Kudela	No^2^	Yes (5 replicates)	Yes	Yes (125)	Yes	Yes	Yes^14^	Yes
*Thalassiosira weissflogii*	Passow	No^1^	Yes (4 replicates)	Yes^5^	No (35)	Yes^7^	Yes	Yes^14^	Yes
*Thalassiosira rotula*	Rynearson	No^3^	Yes (3–5 replicates)	Yes	No (112)	Yes	No^11^	Yes 14	Yes

**A.** Growth rates were determined at a minimum of six temperature conditions. **B.** A minimum of three replicate growth rates were determined. **C.** All other environmental variables were held constant within each individual experiment, other than temperature. These include day length, culture medium, and culture protocols. Saturating nutrients were used to avoid nutrient-induced growth limitation. **D**. Isolates grown at saturating light intensity (µmol quanta m^−2^ s^−1^). **E.** Semi-continuous cultures were diluted using media that was previously adjusted to the appropriate temperature. Dilution frequencies were set so that cells were maintained in constant exponential growth phase and growth rates were reported when cultures were fully acclimated to the experimental conditions, after statistically invariant growth rates were recorded for at least 3–5 generations [98] **F.** Upper and lower thermal limits were tested repeatedly (at least 3 times) **G.** Multiple biomass parameter/proxies were used to determine daily abundance and included cell counts, extracted chlorophyll *a*, and in vivo chlorophyll *a* fluorescence. Each method could be used reliably to determine steady-state acclimation. **H.** At each temperature, the maximum acclimated specific growth rate (d^−1^) for each isolate was determined by regressing the change in the log of fluorescence, cell count or chlorophyll *a* over time and testing the equality of slopes from at least three serial cultures (α = 0.05) [99]. If slopes of serial growth curves were not significantly different, the average regression coefficient was used to estimate the common slope, which represented the average acclimated growth rate and the standard error.

**Footnotes:**

1 Growth rates were determined at five temperatures.

2 Growth rates were determined at 4 temperatures. Cells failed to grow at 35°C and reliable growth estimates could not be obtained at 10°C.

3 Growth rates were determined for 4 temperatures, cells exhibited no growth at 35°C.

4 Isolates also grown in 4 different nitrogen species (nitrate, ammonium, urea, and glutamate).

5 Carbonate system also held within a specific range at ambient conditions.

6 Recorded growth for 5 days after acclimation (not necessarily 3–5 generations).

7 After >8 generations.

8 This was performed for upper limit only.

9 Upper limited tested repeatedly. Lower limit was below 0°C, the lowest temperature tested.

10 Upper and lower limits were tested for *P. donghaiense,* however only lower limit was tested for *Synechococcus*. Upper and lower limit tests were performed twice, not three times.

11 This was performed for upper limit in all isolates, and in one isolate for the lower limit.

12 Fluorescence alone was used.

13 *In vivo* fluorescence reported, but Chl measured at the first and last culture days, as well as PN and PC.

14 Growth rates were determined using *in vivo* fluorescence, but were not significantly different from growth rates determined using cell counts.

15 Significant differences between slopes of replicate cultures were not tested. Instead, the mean slope was used. Variation within a temperature treatment was much smaller than variation between temperatures.

## Discussion

Although we were able to characterise the thermal reaction norms of only a small subset of the resident phytoplankton in the global ocean in this illustrative community-wide experiment, they provide valuable insights into how a warming ocean could influence marine floristics. They also enabled us to conduct the first (as far as we are aware) evaluation of how much confidence we should have in exploiting the rich datasets of the prior physiological literature, and raise some issues about how best to relate such physiological data to the future temperature projections from climate change models. Hence they offer some lessons on where we should focus our efforts in future community-wide experiments.

### Reaction Norm Shape – Implications for Biogeographical Change

The two open ocean end-members are the polar diatoms and tropical diazotrophs, which span a temperature range of 2.4 to 27.5°C that is comparable to that presented in well-cited collations [22], [23]. They represent two groups that play a key role in the biogeochemical cycles of N, C, Fe and Si [47], [48], [49]. The first comparison of reaction norms of unicellular and colonial diazotrophs reveals marked differences between these groups in maximum growth rates and thermal optima that will have implications for future floristic shifts within the diazotroph assemblage (Fu et al., unpublished data). Such a range of temperature reaction norms (Figure 4) supports indirect evidence from oceanographic surveys of the role of environmental conditions in setting the relative distributions of different diazotroph groups [50]. Such floristic shifts (Fig. 3) will certainly have ecological consequences (different pathways for N fixed by uni-cells versus colonial diazotrophs, [8]. Moreover, as thermally-tolerant non-diazotrophic groups may replace N_2_ fixers, as annual temperature ranges begin to exceed the maximum limits of the diazotrophs, there may also be biogeochemical ramifications of such floristic shifts 51,52] (Figure 4). These datasets provide some of the first direct physiological evidence of the potential to alter biome boundaries, as predicted by models based on previously available relatively poor physiological details for different phytoplankton functional groups [53].

Although we have data for only one polar diatom (*P. inermis*, Table 1), the reaction norm reveals that warming could have a detrimental effect on Southern Ocean diatoms by the end of the century if this species is representative (Table 6) as this species would be at or beyond its thermal limit with a 3.5°C rise in temperature (Table 6, Figure 1B). Although there are few prior studies of the temperature ranges of Southern Ocean diatoms [53], at least one study [54] reports a similar growth temperature range of 2 to 9.2°C for a *Chaetoceros* sp. isolated from the Southern Ocean. The reaction norm for the Southern Ocean diatom highlights the lack of oceanic refugia (i.e. colder waters) for these polar species, and raises uncertainties as to whether such a geographically-isolated phytoplankton group (i.e. South of a major physical barrier, the Polar Front) which has a very different photo-physiology [31] can acclimate or adapt on a timescale of decades to warming temperatures. By extension, it is unclear which phytoplankton group(s) might emerge or replace diatoms if they become extinct and how such a floristic shift will affect trophodynamics. Potentially coccolithophores could play expand their range and there is some recent evidence that in the Subantarctic this group is extending their southerly extent [55]. Shifts between diatoms and calcifying phytoplankton could also have major biogeochemical implications for the Southern Ocean Si and C cycles.

**Table 6:** A summary of projected increases in global sea surface temperature for 2020–2029 (relative to 1980 to 1999) and for 2090–2099 (relative to 1980 to 1999) from three IPCC scenarios [104]).

SRES model scenarios	B1 (°C)	A1B (°C)	A2 (°C)
2020–2099	0.8	0.9	1.1
2090–2099	2.1	3.1	3.5

The A2 and A1B scenarios for CO_2_ emissions are very similar to that for observed global emissions [106] and hence were used here for the comparison in Figure 7.

There are major uncertainties in global environmental change research that are difficult to tackle in the confines of laboratory research. For example, far less than 1% of phytoplankton taxa are in culture or culturable and many of those in culture have been cultivated for thousands of generations (decades). Choosing representative species appears daunting as over 40,000 phytoplankton species are described, with thousands more to be discovered [56]. However, by focusing on the major contributors to primary production i.e. phytoplanktonic functional groups in the ocean (cyanobacteria, diatoms, coccolithophorids and dinoflagellates), the list can be narrowed [57]. Within each of those groups, representative species that are important contributors to bloom formation, downward carbon flux, and enhancement of upper ocean N inventories are logical targets for laboratory research. For example, we focused on diatoms that comprise the *Thalassiosira* spp. such as *Thalassiosira rotula*, because they are an important contributor to bloom formation [39], [58] and *Thalassiosira pseudonana,* because it is one of the best-studied physiological model species [37], [59] with a fully sequenced genome [36]. Understanding the growth responses of additional genera that are central to biogeochemical cycling such as the coccolithophores in the genus *Emiliania* and diatoms in the genus *Chaetoceros* will provide important data for understanding phytoplankton response to climate change.

It is also likely that species dominance will alter with changing environmental conditions, leading to the appearance of new and likely unstudied organisms that will need careful physiological characterization. For example, the diatom species *Neodenticula seminae* was sampled in large numbers from North Atlantic waters in 1999 and has become established there over the last decade [60]. This is the first recorded presence of *N. seminae* in the Atlantic in over 800,000 yrs. The “invasion” of this diatom is thought to reflect increasing transport of Pacific waters into North Atlantic waters via the Arctic[60].

One confounding issue of using representative species for phytoplankton functional types is the major differences in reaction norms (Figure 2), between strains for each species we considered. These trends of different thermal reaction norms potentially have parallels in recent studies that report differential responses to CO_2_ enrichment among strains of coccolithophores [61], [62], and diazotrophic cyanobacteria (Hutchins et al. unpublished data). In diatoms, significant inter- and intra-specific variation exists in response to many factors, including light intensity [63], [38], [64]. Our datasets on temperature and diazotrophs suggest that within each genus (i.e. unicellular or colonial), reactions norms differed among strains, albeit to a small degree (Table 3). There were also marked differences in thermal optima between genera, illustrating the dangers of using a sole strain of a cultured phytoplankton species to represent an entire phytoplankton functional group in ecosystem or biogeochemical models. However, we may be more optimistic about parameterizing models at the species level: there is broad similarity in the shape of strains’ reaction norms within a species (Figures 2 and 4, Table 3). This may reflect selection on different taxa to maintain particular ‘shapes’ or underlying constraints in their ability to adapt to different temperature environments. More importantly, it suggests that we may justifiably parameterise biogeochemical models with traits of important species even in the absence of a strong understanding of their intraspecific variation.

### Differential Physiological Responses to Warming between Strains

A further major challenge in global environmental change research is to understand why the reaction norms of multiple strains of the diatoms *Thalassiosira rotula* and *Thalassiosira pseudonana* and the dinoflagellate *A. sanguinea* differed. For example, one of six *Thalassiosira rotula* strains was unable to grow at 4°C and had a significantly higher optimal growth temperature than other strains of *Thalassiosira rotula*. This strain was isolated (∼20 years ago) from the Mediterranean and it is tempting to link its growth response to its provenance. However, the reaction norms of the other two Mediterranean strains tested were more similar to strains sampled from northern temperate regions. This highlights the importance of examining multiple strains from a single region, particularly when testing for region-specific responses to environmental change.

Within species, the variation of growth rates amongst strains changed considerably with temperature. For example, the coefficient of variation (CV) amongst *Thalassiosira rotula* strains was fivefold greater at the low end of its temperature tolerance than at the upper end. In *A. sanguinea,* higher CV’s were observed at the high end of its temperature tolerance than the middle. In general, variation among strains in both diatoms and dinoflagellates was lowest (7–17%) near the optimal growth temperature. This is comparable to previously observed intra-specific CVs of 5–15% in diatoms [63] and 13–39% in dinoflagellates [65] at near-optimal growth temperatures. In contrast, variation amongst strains was often highest at the extremes of temperature tolerance suggesting that genotypic selection pressures would have the largest influence at these points.

In the case of *A. sanguinea*, the temperature-growth response is similar to the trends presented in [66] who reported optimal growth rates at 25°C and a salinity of 20, but with positive growth from 10–30°C and salinities from 10–40. Dinoflagellates are classified as “modified latitudinal cosmopolitan” [67] and true endemism is rare [68]. *A. sanguinea* follows this pattern and is widespread, observed along the west coast of the United States [69], [70], [71], [72], [73], the Gulf of Mexico [74], Brazil [75], Peru [76], Hong Kong [77], Japan [66], Korea [78], and the Black Sea [79]. While several hypotheses have been put forward regarding their range expansion including dispersion of vegetative cells either naturally or by ballast-water transport, there have been no genetic studies to date that provide population structure or gene flow, although microsatellite markers have been developed [80]. While the relatively uniform temperature-growth responses [66], [69] (this study) are consistent with the classification of this species as eurythermal, we note again the strain variability at the low and high end of the temperature range. This strain variability suggests that the widespread success of this organism in neritic waters is due at least in part to substantial strain (genetic) variability within the species. Successful expansion of this genera due to either transport or changes in ocean climate may be largely dependent on the strain(s) present within a given region.

### Comparison of Our Findings to the Temperature Versus Growth Literature

There is a large body of literature collected between 1962–2010 [43], but its value may be limited if there is too much confounding interference due to the differing protocols employed across individual studies. Our datasets provide some preliminary checks and balances to appraise the worth of such prior datasets. The thermal trait analysis reported in [43] differed from that carried out on the species in our study in several ways.

Departures between the literature collation relative to the present study may be related to the quality of the data used in this thermal trait analysis (the literature provides a large repository of data but of more uneven quality relative to the present study) and allows us to characterize areas where efforts such as this study would be most fruitful. For example, we found that differences in optimum temperature for growth between our study and literature estimates were small (Figure 6), suggesting that this trait is robust across a range of experimental methodologies. In contrast, a comparison of the maximum growth rates showed that this trait varied more than for the others examined (Figure 7), likely due to its higher sensitivity to experimental conditions, such as light intensity [69] or composition of lab culture media [81]. Temperature niche width is a more difficult trait to characterize, requiring measurements across a larger range of temperatures than is typically feasible. Therefore, although we see large differences in the distributions of this trait (Figure 5), this is likely due to the high uncertainty in our estimates, rather than absolute differences.

Despite some of the above methodological limitations, these prior published estimates provide a ‘parameter envelope’ reflecting uncertainty introduced through experimental and statistical methods. This uncertainty provides valuable information that can be incorporated when modelling phytoplankton populations [82] and communities [83]. Models focusing on the community level pose the greater challenge, as the uncertainty introduced by measurements made under different environmental conditions must be carefully accounted for (though this may require the collection of additional data). We hope that in future, interactions between temperature and other important parameters (e.g. light, nutrients) will be better characterized, thereby constraining this uncertainty further and improving our ability to model community interactions.

### Trait-environment Relationships

A recent synthesis study [42] reported found that optimum temperature was strongly related to mean environmental temperature, indicative of past adaptation. This pattern was not evident in the present study, probably because of insufficient data from high latitude isolates. In contrast, Thomas et al. did not observe any clear relationship between temperature traits and environmental variability. In our study we do see a positive saturating relationship between temperature niche width and annual temperature range, as predicted by the climate variability hypothesis [84] (see Figure S7). However, intraspecific variation in temperature traits is unrelated to environmental temperature variation and we do not have evidence for local adaptation within a species (see Table 3). This trait-environment relationship is consistent with a simple ecological interpretation: that changes in the temperature variability gradient, rather than local adaptation, drives species turnover. Nevertheless, our study may also lack the power to detect any effects of local adaptation.

Another obvious uncertainty in global environmental change research is the capacity of phytoplankton to evolve new thermal windows, or the lack of such evolutionary resilience [85]. In a thermal experimental evolution study [26], used “ratchet” experiments (incremental increases in growth temperature) to demonstrate differential abilities of phytoplankton groups to adapt to increasing temperature. In general, they found that species from continental lakes were able to adapt to large increases in temperature compared to open ocean phytoplankton groups; for instance, the globally distributed coccolithophore species *Emiliania huxleyi* was one of the species that displayed little or no ability to adapt to warming. Further evolutionary studies in regard to thermal tolerance are called for, including studies focused on the diversity of intraspecific responses. Long term evolutionary inferences can also be made from biogeographical observations, such as the lack of cyanobacteria or coccolithophores in polar marine waters [86]. Evolution of higher temperature tolerance may also be constrained by the existence of correlations and trade-offs among traits and possible opposing selective pressures on correlated traits [87].

Inferences about species range limits can be drawn using their thermal reaction norms. These can be estimated under current and future climate predictions, and aggregated to make predictions about changes to diversity patterns and biogeochemical processes. A recent analysis [43] found that ocean warming this century is likely to lead to a decline in tropical phytoplankton diversity, as many tropical strains, in the absence of evolution, will be unable to survive even small increases in temperature. Merging this approach with ocean biogeochemical models and data on species’ growth vs. nutrient curves will allow us to make even more fine-grained predictions of growth and possibly productivity of different groups in the future. However, evolutionary responses to temperature change need to be accounted for; experiments subjecting different phytoplankton species to different thermal regimes will prove valuable in estimating the rapidity of this process in single species. Future experiments that subject mixed phytoplankton communities to elevated temperature conditions will also be needed, as species interactions can negatively influence both rates of adaptation and productivity [88], [89].

### Merging Physiological Data with Climate Change Model Projections

**Figure 8:**
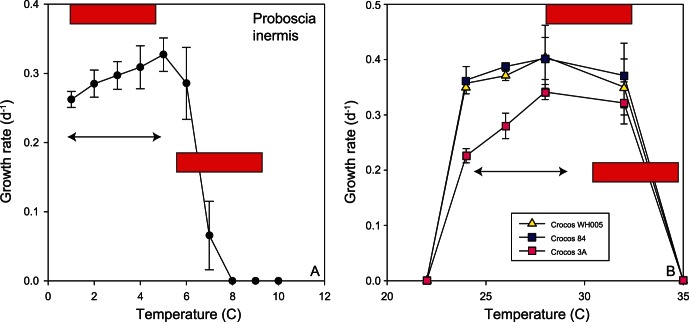
Thermal reaction norms for the two end-members species from our study compared with predicted ocean warming trends. Projected warming by 2020–2029 and 2090–2099 red bars (see Figure 1B) and temperature range (arrow) (from [91]), over the annual cycle is overlaid on the two reaction-norms (0.9 to 4.3°C for the polar diatom and 23.7 to 28.3°C for the *Crocosphaera* strains).

One of our aims was to exploit the growing datasets of climate change projections from models which provide regional or global maps of how ocean conditions, such as temperature, irradiance, nutrient and trace metal supply, pH and carbon dioxide will be altered in the coming decades [2], [90]). In Figure 8 we attempt to merge some of our physiological data for the two thermal end-members – polar diatoms and tropical diazotrophs – with those of model projections for warming from the most realistic IPCC scenario –3.5°C warming by 2100 (Table 6; Figure 1B). This comparison is instructive as it reveals that additional information is required to merge the physiological and the model projections with confidence. The red bars in Figure 8 denote the projected increase in temperature based on the annual minimum and maximum temperature for the regions in which the phytoplankton were isolated [91]. Although information is available regarding the *in situ* temperature at the locales where cells were isolated – for example 3°C for the polar diatom, we do not know whether this species successfully subsists from austral winter to austral summer, or whether it is succeeded by other species over the course of the annual growth season.

Of course, beyond warming effects there are many other uncertainties about how the coastal ocean will respond to climate change. Additional anthropogenic pressures such as eutrophication, acidification, and alterations in salinity and irradiance regimes will all impact HABs and other coastal phytoplankton groups [92]. Consideration of these multivariate influences greatly complicates predictions of the responses of complex natural phytoplankton assemblages to environmental change [86]. In addition, in the laboratory we are limited to examining individual cultured isolates so we have little understanding of how the diverse range of strains/ecotypes present in natural systems might confer some stability or buffer ecosystems against such environmental change [85].

### Next Steps and Lessons Learnt

Although we devoted considerable effort to developing a consensus-based experimental protocol, there were minor departures from it by most participants, that this is one area that can be improved upon. Such improvements, and the development of a more educated community with respect to protocol developments, are essential before we can tackle methodologically more challenging manipulations – trace metals, pH, or manipulation of multiple drivers concurrently, or to increase the number of participating laboratories to >10. This approach builds upon major efforts by the Ocean Acidification community to advocate best methodological practices [93] by being more proactive in developing community-wide protocols. Such community-wide initiatives would benefit from ‘hands-on” workshops similar to that conducted regularly by the Group for Aquatic Primary Productivity (GAP) workshops [94].

In climate change research there is an urgent need to alter what sort of experiments we do – more emphasis should be focused on community-wide experiments (in this short study we conducted measurements of 675 growth rates from 9 species). If we are to develop such community-wide initiatives we must also deal with some of the intrinsic limitations of our existing science culture, in that while collaborative efforts are often warranted and even encouraged, all investigators also have a need to demonstrate their individual productivity and creativity to their peers, their employers, and their funding agencies. Thus, it may be difficult to motivate researchers sufficiently in the long run (i.e. years) to devote considerable effort and scarce funding to obtaining collective datasets. The problem of inadequate motivation to participate in community-based efforts could be partially alleviated if there was a demonstrated interest by funding agencies in funding for and recognition of the needs and benefits of such community-wide research. These types of funding issues are especially problematic for international collaborative groups, due to the lack of coordination and communication between national funding agencies. These pressing issues and impasses have also been recognised recently for the field of macro-ecology [95].

## Materials and Methods

### Rationale for Selection of Temperature Versus Growth Rate

We designed a community-wide experiment examining the effect of temperature on phytoplankton growth for two general reasons. First, there is a high degree of consensus that there will be significant changes in temperature based on projections from climate change models (relative to other environmental properties such as incident irradiance or trace metal supply where changes are more uncertain, [86]. Second, it is relatively straightforward to manipulate temperature in laboratory experiments using cultured phytoplankton isolates (versus pH or CO_2_, [96], [93] and hence this variable is more amenable to developing and implementing a common protocol for community-wide experiments. In many cases, temperature is considered to be a master variable or ultimately limiting property for phytoplankton growth [23], [26].

The choice of growth rate as a physiological metric to measure the effect of temperature was selected because it is fundamental to the success of phytoplankton species and it ultimately integrates phytoplankton physiology [37], since for an asexually reproducing unicellular organism it represents the most direct measure of reproductive success and therefore of fitness in the environment. By directly measuring fitness (e.g. growth rate) over a range of temperatures, a reaction norm can be described for each genotype (culture isolate) with respect to temperature [97]. Ultimately, one would describe reaction norms for each physiological variable under a range of environmental conditions to understand both the direct and interactive effects of factors that affect phytoplankton growth.

### Phytoplankton Species

The species used for this community-wide experiment in some cases were those already being maintained in culture for other experimental studies by each participating lab. They included both eukaryotes (diatoms and dinoflagellates) and prokaryotes (cyanobacteria) collected from a range of environments including polar, temperate and tropical waters (Figure 1A). A summary of the provenance, distribution and environmental relevance of each species/strain is provided in Table 1. For five species, up to six strains were tested to get an estimate of intraspecific variation in thermal traits (Table 2).

### Lab Culturing Protocol

The following protocol was agreed upon by the participating labs and generally implemented here. Inevitably there were minor departures from this consensus-based protocol which are detailed in Table 5. These departures were largely due to laboratory-specific logistics and provide insights as to how future community-wide manipulation experiments can be improved upon.

1. For each isolate, growth rates were determined at a minimum of six temperatures for each growth curve.
2. A minimum of three replicate growth rates were determined at each temperature.
3. All other environmental variables were held constant within each individual experiment, other than temperature. These include light intensity, day length, culture medium and culture protocols. Saturating nutrient concentrations were used in all experiments to avoid nutrient-induced growth limitation. Light intensity varied among experiments but was most often at saturating levels (determined based on a range of approaches including PE curves and prior investigation of irradiance versus growth responses) for the particular culture isolate (Table 5). Each laboratory used protocols that were most appropriate for the cultured isolates they were working with. A description of culturing conditions for each species is provided in Table 5.
4. Semi-continuous cultures were diluted using media that had been pre-conditioned to the appropriate temperature. Dilution frequencies were set so that cells were maintained in exponential growth phase and the growth rates reported are for cultures fully acclimated to the experimental conditions, (e.g. after statistically invariant growth rates were recorded for at least 4 generations [98]).
5. The upper and lower thermal limits for each species/strain were determined by repeatedly (i.e. up to three times) incubating cultures at temperatures at which they did not grow (see Discussion).
6. Multiple biomass parameters/proxies were used to determine growth rates. These included: cell counts, extracted chlorophyll *a*, and *in vivo* chlorophyll *a* fluorescence. Each method reliably estimated biomass and growth rates, and was used to ascertain when steady-state growth rates were achieved, and to monitor the acclimation of the cultures as outlined above.
7. At each temperature, the mean steady-state specific growth rate (d^−1^) for each isolate acclimated for at least four generations, was determined by regressing and testing the equality of slopes (i.e. temporal change in the log of fluorescence, cell count or chlorophyll *a*) from at least three serial cultures (α = 0.05) [99]. If the slopes of serial growth curves were not significantly different, the average regression coefficient was used to estimate the common slope, which represented the average steady-state growth rate in cultures acclimated to a specific temperature and the standard error. We did not impose a standard method to generate the temperature growth rate response curves. The individual methods used are presented in Table 5.
8. For each species where multiple strains were examined, analysis of variance was used to test for intraspecific differences in growth rates at each temperature examined (α = 0.05) and the coefficient of variation (CV) was calculated to determine the extent of growth rate variation among strains. Statistical analyses were performed in SPSS V19 (IBM, Inc).

### Estimation of the Thermal Reaction Norm Parameters

We used the following equation (modified from [100]) to describe the thermal reaction norms of each strain:
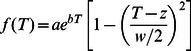
(1)where specific growth rate *f* depends on temperature, *T*, as well as parameters *z*, *w*, *a*, and *b*. *w* is the temperature niche width, while the other three possess no explicit biological meaning. We fit (1) to the growth data for each strain using maximum likelihood to obtain estimates for parameters *z*, *w*, *a* and *b*. In addition, we estimated the optimum temperature for growth and maximum growth rate by numerically maximizing the equation after estimating the parameter values. Note, estimates of temperature niche width for three *T. pseudonana* strains (CCMP 1014, 1015 & 1335) and one strain of *A. sanguinea* (YRB) are inflated because of poor resolution of the lower limit for growth (see Results).

### Thermal Trait Analyses

These analyses focused on three traits that describe the thermal tolerance curve: optimum temperature for growth, temperature niche width and maximum growth rate.

#### Comparison with literature traits

We tested for heterogeneity in intraspecific variance of all three traits using Levene’s test centered by the median [101]. We also compared the estimated species traits to those estimated from previously published thermal reaction norms of the same species. These data were collated from ten publications (Appendix S1). In cases where different prior published studies measured growth rates on the same strain, we pooled the measurements before estimating the thermal reaction norm parameters. This has the drawback of ignoring interactive effects caused by differences in experimental conditions between previous studies, but was necessary for two reasons. Firstly, in several prior studies only growth rates below the optimum were measured and these data could not be used without pooling, and secondly we have a poor understanding of how the differences in experimental conditions (irradiance, day length, nutrient concentrations, etc.) interact with temperature to determine growth rate, preventing us from incorporating covariates in any meaningful way.

#### Testing for interspecific differences in thermal traits

We also tested whether there were significant differences among species in thermal trait distributions. In order to account for uncertainty in our estimates of these traits, we used a parametric bootstrapping approach coupled with information criterion-based model selection [102]. This comparison was performed on the five species for which we measured more than a single strain (*Thalassiosira pseudonana*, *Thalassiosira rotula*, *T. erythraeum*, *A. sanguinea* and *C. watsonii*). For each strain, we fitted the function described earlier to the growth rate measurements and extracted the residuals from this fit. We then performed 1000 residual bootstraps, a procedure in which the residuals are randomly ‘reassigned’ to predicted values (each of which corresponds to a growth rate measurement) and added to them, thereby generating a slightly different thermal reaction norm [103]. For each iteration, we refitted the function and estimated the parameters (*z, w, a, b*) and also the two derived traits, maximum growth rate and optimum temperature for growth. Examining the distribution of these parameters and traits over the 1000 bootstraps allows us to quantify the uncertainty in our estimates, which we can then incorporate in models seeking to explain variation in them.

For each set of bootstraps of all 21 strains (i.e. a relatively small dataset for interspecific comparisons), we fitted two linear models to each trait (optimum temperature, temperature niche width and maximum growth rate) and compared their explanatory ability using AICc values (Akaike Information Criterion, corrected for small sample size) models rather than AIC. One of these models included just an intercept term while the other had both an intercept and species identity as an explanatory variable. We then examined the distribution of delta AICc (AICc [model with species identity] minus AICc [model without]) values over the 1000 bootstraps to determine whether species identity was a useful predictor of each of the three thermal traits. If the entire 95% confidence interval of AICc values exceeded zero, we concluded that species identity was a useful predictor and that species differed in the distribution of the trait.

#### Comparison of traits with environmental data

We obtained estimates for mean annual and monthly mean temperatures at locations close to the isolation location of each strain using the World Ocean Atlas [91]. We approximated the annual temperature ranges for each species using the difference between the maximum monthly mean temperature and the minimum monthly mean temperature at each location, i.e. thermal variability *sensu* [46]. Thereafter, we attempted to explain variation in three temperature traits (optimum temperature for growth, temperature niche width, and maximum growth rate) with linear models containing these environmental parameters for each species. The best model was chosen using AIC values. Statistical analyses were performed using a combination of least squares and maximum likelihood estimation techniques using the R statistical computing environment (version 2.15.0).

## Supporting Information

Figure S1 **The individual reactions norms (specific growth rates per day) of all measured strains and cultures obtained by fitting a thermal tolerance function (see Methods) to these data.**
(TIF)

Figure S2 **Intraspecific variation in thermal reaction norms for species in which only one strain was available.**
(TIF)

Figure S3 **A summary of temperature optima, maximum growth rates and niche width – expressed as box and whiskers plots - for each of the species used in our study.** The black bands denote the median value, the bottom and top of the box represent the 1st and 3rd quartile of the data, respectively. The ‘whiskers’ extending from the boxes indicate the positions of the lowest & highest values in the data. If the sample size is small enough, the whiskers may not appear (e.g. if there are only 3 equally spaced points, the value represented as the 1st quartile is the lowest value).
(TIF)

Figure S4 **A summary of temperature optima (°C) obtained here (red boxes) and from the literature (blue boxes), expressed as box and whiskers plots, for each of the species used in our study (red).** The thick black line in each box represents the median temperature.
(TIF)

Figure S5 **A summary of niche width (°C), expressed as box and whiskers plots - for each of the species used in our study.**
(TIF)

Figure S6 **A summary of maximum growth rate expressed as box and whiskers plots - for each of the species used in our study.**
(TIF)

Figure S7 **A plot of niche widths versus annual temperature range.** Data points are coloured by species. This plot omits the niche widths that are poorly resolved (i.e. the 6 *T. pseudonana* +1 *A. sanguinea* strain). Niche widths increase as the annual temperature range increases, in accordance with the climate variability hypothesis.
(TIF)

Appendix S1 (DOCX)

## References

[1] Bopp L, Monfray P, Aumont O, Dufresne JL, Treut H, et al (2001) Potential Impact of Climate Change on Marine Export Production. Glob Biogeochem Cy 15: 81–99 doi:10.1029/1999GB001256.

[2] Sarmiento JL, Slater R, Barber R, Bopp L, Doney SC, et al (2004) Response of ocean ecosystems to climate warming. Glob Biogeochem Cy 18: GB3003.

[3] Chevin LM, Lande R, Mace GM (2010) Adaptation, plasticity, and extinction in a changing environment: towards a predictive theory. PLoS Biol 8: e1000357. 20463950 10.1371/journal.pbio.1000357 PMC2864732

[4] [4. Moore JK, Doney SC, Glover DM, Fung IY (2002) Iron cycling and nutrient limitation patterns in surface waters of the world ocean. Deep-Sea Res II 49: 463–508.

[5] Field CB, Behrenfeld MJ, Randerson JT, Falkowski P (1998) Primary production of the biosphere: integrating terrestrial and oceanic components. Science 281: 237–240. 9657713 10.1126/science.281.5374.237

[6] Behrenfeld MJ, Worthington K, Sherrell RM, Chavez FP, Strutton P, et al (2006) Controls on tropical Pacific Ocean productivity revealed through nutrient stress diagnostics. Nature 442: 1025–1028. 16943835 10.1038/nature05083

[7] Hutchins DA, Mulholland MR, Fu FX (2009) Nutrient cycles and marine microbes in a CO_2_-enriched ocean. Oceanogr 22: 128–145.

[8] Mulholland MR (2007) The fate of nitrogen fixed by diazotrophs in the ocean. Biogeosci 4: 37–51.

[9] Boyd PW, Doney SC (2003) The impact of climate change and feedback processes on the Ocean Carbon Cycle. In: Fasham MJR editor. Ocean Biogeochemistry – the role of the ocean carbon cycle in global change. Springer-Verlag, Berlin. pp. 157–187.

[10] Sarmiento JL, Hughes TMC, Stouffer RJ, Manabe S (1998) Simulated response of the ocean carbon cycle to anthropogenic climate warming. Nature 393: 245–249.

[11] Levitus S, Antonov JI, Boyer TP, Locarnini RA, Garcia HE, et al (2009) Global ocean heat content 1955–2008 in light of recently revealed instrumentation problems, Geophys Res Lett. 36: L07608 doi:10.1029/2008GL037155.

[12] Bakun A, Field DB, Redondo-Rodriguez A, Weeks SJ (2010) Greenhouse gas, upwelling-favorable winds, and the future of coastal ocean upwelling ecosystems. Glob Change Biol 16: 1213–1228.

[13] Raven JA, Geider RJ (1988) Temperature and algal growth. New Phytol 110: 441–461 doi:10.1111/j.1469-8137.1988.tb00282.x.

[14] Moisan JR, Moisan TA, Abbot MR (2002) Modelling the effect of temperature on the maximum growth rates of phytoplankton populations. Ecol Model 153: 197–215.

[15] Longhurst AR (1998) Ecological geography of the sea. Academic Press. 527p.

[16] Needoba JA, Foster RA, Sakamoto C, Zehr JP, Johnson KS (2007) Nitrogen fixation by unicellular cyanobacteria in the temperate oligotrophic North Pacific Ocean. Limnol Oceanogr 52: 1317–1327.

[17] Behrenfeld MJ, Falkowsi PG (1997) A consumer’s guide to phytoplankton primary productivity models. Limnol Oceanogr 42: 1479–1491.

[18] Fu FX, Warner ME, Zhang Y, Feng Y, Hutchins DA (2007) Effects of increased temperature and CO_2_ on photosynthesis, growth and elemental ratios of marine *Synechococcus* and *Prochlorococcus* (Cyanobacteria). J Phycol 43: 485–496.

[19] Hare C, Leblanc K, DiTullio GR, Kudela KM, Zhang Y, et al (2007) Consequences of increased temperature and CO_2_ for phytoplankton community structure in the Bering Sea. Mar Ecol Prog Ser 352: 9–16.

[20] Rose JM, Feng Y, DiTullio GR, Dunbar RB, Hare CE, et al (2009) Synergistic effects of iron and temperature on Antarctic phytoplankton and microzooplankton assemblages. Biogeosci 6: 3131–3147.

[21] Feng Y, Hare CE, Leblanc K, Rose JM, Zhang Y, et al (2009) Effects of increased pCO_2_ and temperature on the North Atlantic spring bloom. I. The phytoplankton community and biogeochemical response. Mar Ecol Prog Ser 388: 13–25.

[22] Banse K (1991) Rates of Phytoplankton Cell Division in the Field and in Iron Enrichment Experiments. Limnol Oceanogr 36: 1886–1898.

[23] Eppley RW (1972) Temperature and phytoplankton growth in the sea. Fish Bull 70: 1063–1085.

[24] Li WKW (1985) Photosynthetic response to temperature of marine phytoplankton along a latitudinal gradient (16°N to 74°N). Deep Sea Res 32: 1381–1385.

[25] Doney SC, Lindsay K, Caldeira K, Campin J-M, Drange H, et al (2004) Evaluating global ocean carbon models: the importance of realistic physics, Glob Biogeochem Cy. 18: GB3017 doi:10.1029/2003GB002150.

[26] Huertas IE, Rouco M, López-Rodas V, Costas E (2011) Warming will affect phytoplankton differently: evidence through a mechanistic approach. Proc R Soc B 278: 3534–3543 doi:10.1098/rspb.2011.0160. 10.1098/rspb.2011.0160 PMC3189365 21508031

[27] Sarmento H, Montoya JM, Vazquez-Dominguez E, Vaque D, Gasol JM (2010) Warming effects on marine microbial foodweb processes: how far can we go when it comes to predictions? Phil Trans R Soc B 365: 2137–2149. 20513721 10.1098/rstb.2010.0045 PMC2880134

[28] www.galaxyzoo.org. Accessed 2013 April. See also doi; available: 10.1111/j.1365-2966.2010.17432.

[29] Khatib F, DiMaio F, Foldit Contenders Group; Foldit Void Crushers Group, Cooper S, et al. (2011) Crystal structure of a monomeric retroviral protease solved by protein folding game players. Nature Struct Molec Biol http://dx.doi.org/10.1038/nsmb.2119. 10.1038/nsmb.2119 PMC3705907 21926992

[30] Strzepek RF, Maldonado MT, Hunter KA, Frew RD, Boyd PW (2011) Adaptive strategies by Southern Ocean phytoplankton to lessen iron limitation: Uptake of organically complexed iron and reduced cellular iron requirements. Limnol Oceanogr 56: 1983–2002.

[31] Strzepek RF, Hunter KA, Frew RD, Harrison PJ, Boyd PW (2012) Absence of iron-light co-limitation in Southern Ocean phytoplankton. Limnol Oceanogr 57: 1182–1200.

[32] Hu Z, Xu N, Duan S, Mulholland MR (accepted) Growth and nitrogen uptake kinetics for *Prorocentrum donghaiense* acclimated to different nitrogen sources. Harmful Algae (in press).

[33] Najjar R, Pyke CR, Adams MB, Breitburg D, Hershner C, et al (2010) Potential climate-change impacts on the Chesapeake Bay. Est Coast Shelf Sci 86: 1–20.

[34] Guillard RRL, Ryther JH (1962) Studies of marine planktonic diatoms I. *Cyclotella nana* Hustedt, and *Detonula confervacea* (Cleve) Gran. Can J Microbiol 8: 229–239. 13902807 10.1139/m62-029

[35] Sakshaug E, Demers S, Yentsch CM (1987) *Thalassiosira oceanica* and *T. pseudonana*: Two different photoadaptational responses, Mar Ecol Prog Ser. 41 275–282.

[36] Armbrust EV, Berges JA, Bowler C, Green BR, Martinez D, et al (2004) The genome of the diatom *Thalassiosira pseudonana*: Ecology, evolution, and metabolism. Science 306: 79–86. 15459382 10.1126/science.1101156

[37] Sunda WG, Huntsman SA (1995) Iron uptake and growth limitation in oceanic and coastal phytoplankton. Mar Chem 50: 189–206.

[38] Strzepek RF, Harrison PJ (2004) Photosynthetic architecture differs in coastal and oceanic diatoms. Nature 431: 689–692. 15470428 10.1038/nature02954

[39] Smayda TJ (1958) Biogeographical studies of marine phytoplankton. Oikos 9: 158.

[40] Krawiec RW (1982) Autecology and clonal variability of the marine centric diatom *Thalassiosira rotula*; (Bacillariophyceae) in response to light, temperature and salinity. Mar Biol 69: 79–89.

[41] Zehr JP, Waterbury JB, Turner PJ, Montoya JP, Omoregie E, et al (2001) Unicellular cyanobacteria fix N_2_ in the subtropical North Pacific Ocean. Nature 412: 635–638. 11493920 10.1038/35088063

[42] Tortell PD, Payne C, Gueguen C, Strzepek RF, Boyd PW, et al (2008) Inorganic carbon uptake by Southern Ocean phytoplankton. Limnol Oceanogr 53: 1266–1278.

[43] Thomas MK, Kremer CT, Klausmeier CA, Litchman E (2012) A global pattern of thermal adaptation in marine phytoplankton. Science, DOI: 10.1126/science.1224836. 10.1126/science.1224836 23112294

[44] Hoppe HG, Gocke K, Koppe R, Begler C (2002) Bacterial growth and primary production along a north-south transect of the Atlantic Ocean. Nature 416: 168–171. 11894092 10.1038/416168a

[45] Taucher J, Oschlies A (2011) Can we predict the direction of marine primary production change under global warming? Geophys Res Lett 38: L02603 doi:10.1029/2010GL045934.

[46] Tuck C, Romanuk TN (2012) Robustness to thermal variability differs along a latitudinal gradient in zooplankton communities. Glob Change Biol 18: 1597–1608 doi:10.1111/j.1365-2486.2012.02652.x.

[47] Hutchins DA, Fu FX (2008) Linking the oceanic biogeochemistry of iron and phosphorus with the marine nitrogen cycle. In: Capone DG, Bronk DA, Mulholland MR, Carpenter EJ, editors. Nitrogen in the Marine Environment, 2^nd^ Edition.Elsevier Press, Amsterdam. 1627–1653.

[48] Treguer P, Nelson DM, van Bennekom AJ, Demaster DJ, Leynaert A, et al (1995) The silica balance in the world ocean: A re-estimate. Science 268: 375–379. 17746543 10.1126/science.268.5209.375

[49] Blain S, Queguiner B, Armand L, Belviso S, Bombled B, et al (2007) Effect of natural iron fertilization on carbon sequestration in the Southern Ocean. Nature 446: 1070–1074. 17460670 10.1038/nature05700

[50] Kitajima K, Furuya K, Hashihama F, Takeda S, Kanda J (2009) Latitudinal distribution of diazotrophs and their nitrogen fixation in the tropical and subtropical western North Pacific. Limnol Oceanog. 54: 537–547.

[51] Breitbarth E, Oschlies A, LaRoche J (2007) Physiological constraints on the global distribution of *Trichodesmium* – effect of temperature on diazotrophy. Biogeosci 4: 53–61.

[52] Boyd PW, Hutchins DA (2012) Understanding the responses of ocean biota to a complex matrix of cumulative anthropogenic change. Mar Ecol Prog Ser 470: 125–135.

[53] Boyd PW, Doney SC (2002) Modelling regional responses by marine pelagic ecosystems to global climate change. Geophys Res Lett 29: DOI: 10.1029/2001GL014130.

[54] Reay DS, Nedwell DB, Priddle J, Ellis-Evans JC (1999) Temperature Dependence of Inorganic Nitrogen Uptake: Reduced Affinity for Nitrate at Suboptimal Temperatures in Both Algae and Bacteria. Appl Environ Microbiol 65: 2577–2584. 10347046 10.1128/aem.65.6.2577-2584.1999 PMC91381

[55] Cubillos JC, Wright SW, Nash G, de Salas MF, Griffiths B, et al (2007) Shifts in geographic distributions of calcification morphotypes of the coccolithophorid *Emiliania huxleyi* in the Southern Ocean during 2001–2006. Mar Ecol Prog Ser 348: 47–58.

[56] Andersen RA (1992) Diversity of eukaryotic algae. Biodiv Conserv 1: 267–292.

[57] Hood RR, Laws EA, Armstrong RA, Bates NR, Browne CW, et al (2006) Pelagic functional group modeling: Progress, challenges and prospects. Deep-Sea Res II 53: 459–512.

[58] Pratt DM (1959) The phytoplankton of Narragansett Bay. Limnol Oceanogr 4: 425.

[59] Sunda WG, Huntsman SA (1997) Interrelated influence of iron, light and cell size on marine phytoplankton growth. Nature 390: 389–392.

[60] Reid PC, Johns DG, Edwards M, Starr M, Poulin M, et al (2007) A biological consequence of reducing Arctic ice cover: arrival of the Pacific diatom *Neodenticula seminae* in the North Atlantic for the first time in 800,000 years. Glob Change Biol 13: 1910–1921.

[61] Langer G, Geisen M, Baumann KH, Klas J, Riebesell U, et al (2006) Species-specific responses of calcifying algae to changing seawater carbonate chemistry. Geochem Geophys Geosyst 7: Q09006.

[62] Langer G, Nehrke G, Probert I, Ly J, Ziveri P (2009) Strain-specific responses of *Emiliania huxleyi* to changing seawater carbonate chemistry. Biogeosci 6: 2637–2646.

[63] Rynearson TA, Armbrust EV (2004) Genetic differentiation among populations of the planktonic marine diatom *Ditylum brightwellii* (Bacillariophyceae). J Phycol 40: 34–43.

[64] Whittaker K, Rignanese D, Olson R, Rynearson T (2012) Molecular subdivision of the marine diatom *Thalassiosira rotula* and its relationship to differences in geographic distribution, genome size, and physiology. BMC Evol Biol 12: 209–217. 23102148 10.1186/1471-2148-12-209 PMC3544637

[65] Costas E (1990) Genetic variability in growth rates of marine dinoflagellates. Genetica 82: 99–102.

[66] Matsubara T, Nagasoe S, Yamasaki S, Shikata T, Shimasaki Y, et al (2007) Effects of temperature, salinity, and irradiance on the growth of the dinoflagellate *Akashiwo sanguinea* . J Exp Mar Biol Ecol 342: 226–230.

[67] Taylor FJR (1987) Dinoflagellate ecology: general and marine ecosystems. In: Taylor FJR (Ed.), The biology of dinoflagellates. Bot Monogr 21: 398–502.

[68] Taylor FJR, Hoppenrath M, Saldarriaga JF (2008) Dinoflagellate diversity and distribution. Biodiv & Conserv 17: 477–418.

[69] Thomas WH, Dodson AN, Linden CA (1973) Optimum light and temperature requirements for *Gymnodinium splendens*, a larval fish food organism. Fish Bull 71: 599–601.

[70] Horner RA, Garrison DL, Plumley FG (1997) Harmful algal blooms and red tide problems on the U.S. west coast. Limnol Oceanogr 42: 1076–1088.

[71] Jessup DA, Miller MA, Ryan JP, Nevins HM, Kerkering HA, et al (2009) Mass stranding of marine birds caused by a surfactant-producing red tide. PLoS ONE 4: e4550. 19234604 10.1371/journal.pone.0004550 PMC2641015

[72] Cloern JE, Schraga TS, Lopez CB, Knowles N, Labiosa RG, et al (2005) Climate anomalies generate an exceptional dinoflagellate bloom in San Francisco Bay. Geophys Res Lett 32: L14608.

[73] Du X, Peterson W, McCulloch A, Liu G (2011) An unusual bloom of the dinoflagellate *Akashiwo sanguinea* off the central Oregon, USA, coast in autumn 2009, Harmful Algae. 10: 784–793.

[74] Robichaux RJ, Dortch Q, Wrenn JH (1988) Occurrence of *Gymnodinium sanguineum* in Louisiana and Texas coastal waters, 1989–1994. In: Zimmerman R, editor. Characteristics and causes of Texas marine strandings, pp19–26, NOAA Tech. Rep. NMFS 143, Washington.

[75] Domingos P, Menezes M (1998) Taxonomic remarks on plankton phytoflagellates in a hypertrophic tropical lagoon (Brazil). Hydrobiol 369/370: 297–313.

[76] Trainer VL, Pitcher GC, Reguera B, Smayda TJ (2010) The distribution and impacts of harmful algal bloom species in eastern boundary upwelling systems. Prog Oceanogr 85: 33–52.

[77] Lu SH, Hodgkiss IJ (2004) Harmful algal bloom causative collected from Hong Kong coastal waters. Hydrobiol 512: 231–238.

[78] Lee CK, Lee OH, Lee SM (2005) Impacts of temperature, salinity and irradiance on the growth of ten harmful algal bloom-forming microalgae isolated in Korean coastal waters. The Sea (J Korean Soc Oceanogr) 10: 79–91.

[79] Gómez F, Boicenco F (2004) An annotated checklist of dinoflagellates in the Black Sea. Hydrobiol 517: 43–59.

[80] Cho SY, Nagai S, Nishitani G, Han MS (2009) Development of compound microsatellite markers in red-tide-causing dinoflagellate *Akashiwo sanguinea* (Dinophyceae). Mol Ecol Resources 9: 915–917. 10.1111/j.1755-0998.2008.02474.x 21564789

[81] Timmermans KR, Wagt BVD, Veldhuis MJW, Maatman A, de Baar HJW (2005) Physiological responses of three species of marine pico-phytoplankton to ammonium, phosphate, iron and light limitation, J Sea Res. 53: 109–120.

[82] Mehnert G, Leunert F, Ciré S, Johnk KSD, Rucker J, et al.. (2010) Competitiveness of invasive and native cyanobacteria from temperate freshwaters under various light and temperature conditions. J Plank Res 32, 1009–1021.

[83] Follows MJ, Dutkiewicz S, Grant S, Chishom SW (2007) Emergent Biogeography of Microbial Communities in a Model Ocean. Science 315: 1843–1846. 17395828 10.1126/science.1138544

[84] Stevens GC (1989) The latitudinal gradient in geographical range: How so many species coexist in the tropics, Amer Naturalist. 133: 240–256.

[85] Reusch TBH, Boyd PW (2013) Experimental Evolution meets marine phytoplankton. Evolution, DOI: 10.1111/evo.12035. 10.1111/evo.12035 23815643

[86] Boyd PW, Strzepek R, Fu FX, Hutchins DA (2010) Environmental control of open-ocean phytoplankton groups: now and in the future. Limnol Oceanogr 55: 1353–1376.

[87] Etterson JR, Shaw RG (2001) Constraint to adaptive evolution in response to global warming. Science 294: 151–154. 11588260 10.1126/science.1063656

[88] Litchman E, Edwards KF, Klausmeier CA, Thomas MK (2012) Phytoplankton niches, traits and eco-evolutionary responses to global environmental change. Mar Ecol Prog Ser 470, 235–248.

[89] Collins S (2011) Competition limits adaptation and productivity in a photosynthetic alga at elevated CO_2_ . Proc Roy Soc B Biol Sci 278: 247–255. 10.1098/rspb.2010.1173 PMC3013392 20685702

[90] Boyd PW, Doney SC, Strzepek R, Dusenberry J, Lindsay K, et al (2008) Climate-mediated changes to mixed-layer properties in the Southern Ocean: assessing the phytoplankton response. Biogeosci 5: 847–864.

[91] Locarnini RA, Mishonov AV, Antonov JI, Boyer TP, Garcia HE, et al. (2010) World Ocean Atlas 2009, Volume 1: *Temperature.* Levitus S, editor. NOAA Atlas NESDIS 68, U.S. Government Printing Office, Washington, D.C., 184 p.

[92] Fu FX, Tatters AO, Hutchins DA (2012) Global change and the future of harmful algal blooms in the ocean. Mar Ecol Prog Ser 470: 207–233.

[93] Riebesell U, Fabry VJ, Hansson L, Gattuso JP (2010) editors. Guide to best practices for ocean acidification research and data reporting, 260 p. Luxembourg: Publications Office of the European Union.

[94] MacIntyre H, Berman T, Berman Frank I (2009) Progress and perspectives in aquatic primary productivity: highlights of the GAP VIII workshop, Eilat, Israel, 2008. Aquat Microb Ecol 56, 109–111.

[95] Nogués-Bravo D, Rahbek C (2011) Communities Under Climate Change. Science 334: 1070 DOI: 10.1126/science.1214833. 22116871 10.1126/science.1214833

[96] Hurd CL, Hepburn CD, Currie KI, Raven JA, Hunter KA (2009) Testing the effects of ocean acidification on algal metabolism: considerations for experimental designs. J Phycol 45: 1236–1251. 27032579 10.1111/j.1529-8817.2009.00768.x

[97] Stearns SC (1989) The evolutionary significance of phenotypic plasticity. Biosci 39: 436–445.

[98] Brand LE, Guillard RRL, Murphy LS (1981) A method for the rapid and precise determination of acclimated phytoplankton reproduction rates. J Plank Res 3: 193–201.

[99] Rynearson TA, Armbrust EV (2000) DNA fingerprinting reveals extensive genetic diversity in a field population of the centric diatom *Ditylum brightwellii* . Limnol Oceanogr 45: 1329–1340.

[100] Norberg J (2004) Biodiversity and ecosystem functioning: A complex adaptive systems approach. Limnol Oceanogr 49; 1269–1277.

[101] Brown MB, Forsythe AB (1974) Robust tests for the equality of variances. J American Stat Assoc 69: 364–367.

[102] Burnham KP, Anderson DR (2002) Model selection and multimodel inference: A practical information-theoretic approach. Springer-Verlag, New York, ed. 2^nd^. 488 p.

[103] Hesterberg T, Moore DS, Monaghan S, Clipson A, Epstein R, et al.. (2010) Bootstrap Methods and Permutation Tests, Chapter 16 for Introduction to the Practice of Statistics, 7th edition, Moore DS, McCabe GP, and Craig BA, editors. W. H. Freeman, N.Y.

[104] Solomon S, Qin D, Manning M, Chen M, Marquiset M, et al.. (2007) Climate Change 2007: The Physical Science Basis, Contribution of Working Group I to the Fourth Assessment Report of the Intergovernmental Panel on Climate Change, Cambridge University Press, ISBN 978-0-521-88009-1 (pb: 978-0-521-70596-7).

[105] Phillips EM, Zamon JE, Nevins HM, McGibble CM, Duerr RS, et al (2011) Summary of birds killed by a harmful algal bloom along the south Washington and north Oregon coasts during October 2009. Northwestern Naturalist 92: 120–126.

[106] Raupach MR, Marland G, Ciais P, Le Quere C, Canadell JG, et al (2007) Global and regional drivers of accelerating CO_2_ emissions. PNAS 104: 10288–10293. 17519334 10.1073/pnas.0700609104 PMC1876160

