# Supplementary figures and images for: Marine Phytoplankton Temperature versus Growth Responses from Polar to Tropical Waters – Outcome of a Scientific Community-Wide Study
Source: PLoS One. 2013 May 21;8(5):e63091. doi: 10.1371/journal.pone.0063091 (PMC3660375; doi:10.1371/journal.pone.0063091)

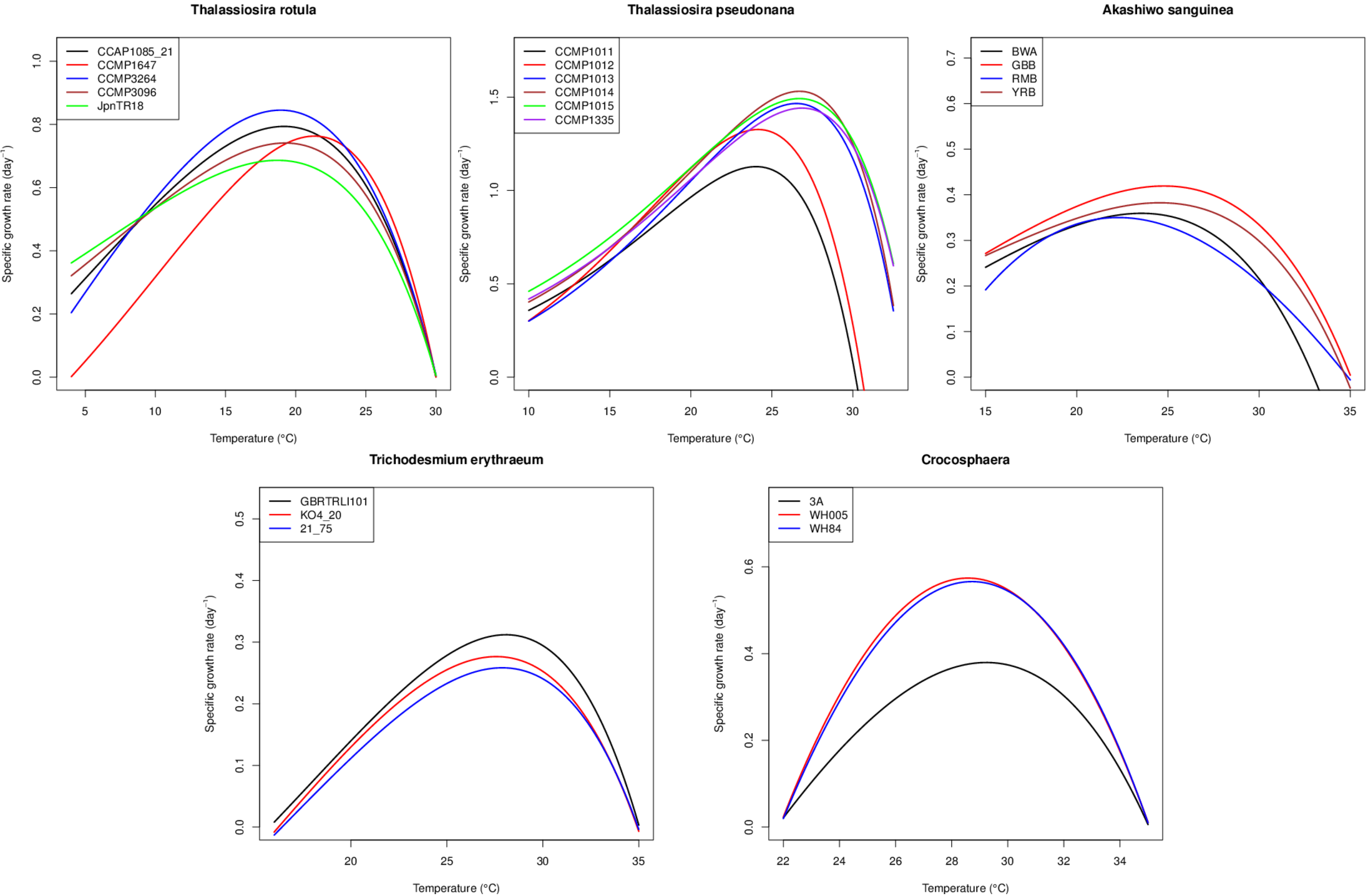

Supplement: Figure S1 — The individual reactions norms (specific growth rates per day) of all measured strains and cultures obtained by fitting a thermal tolerance function (see Methods) to these data. (TIF) [file pone.0063091.s001.tif]

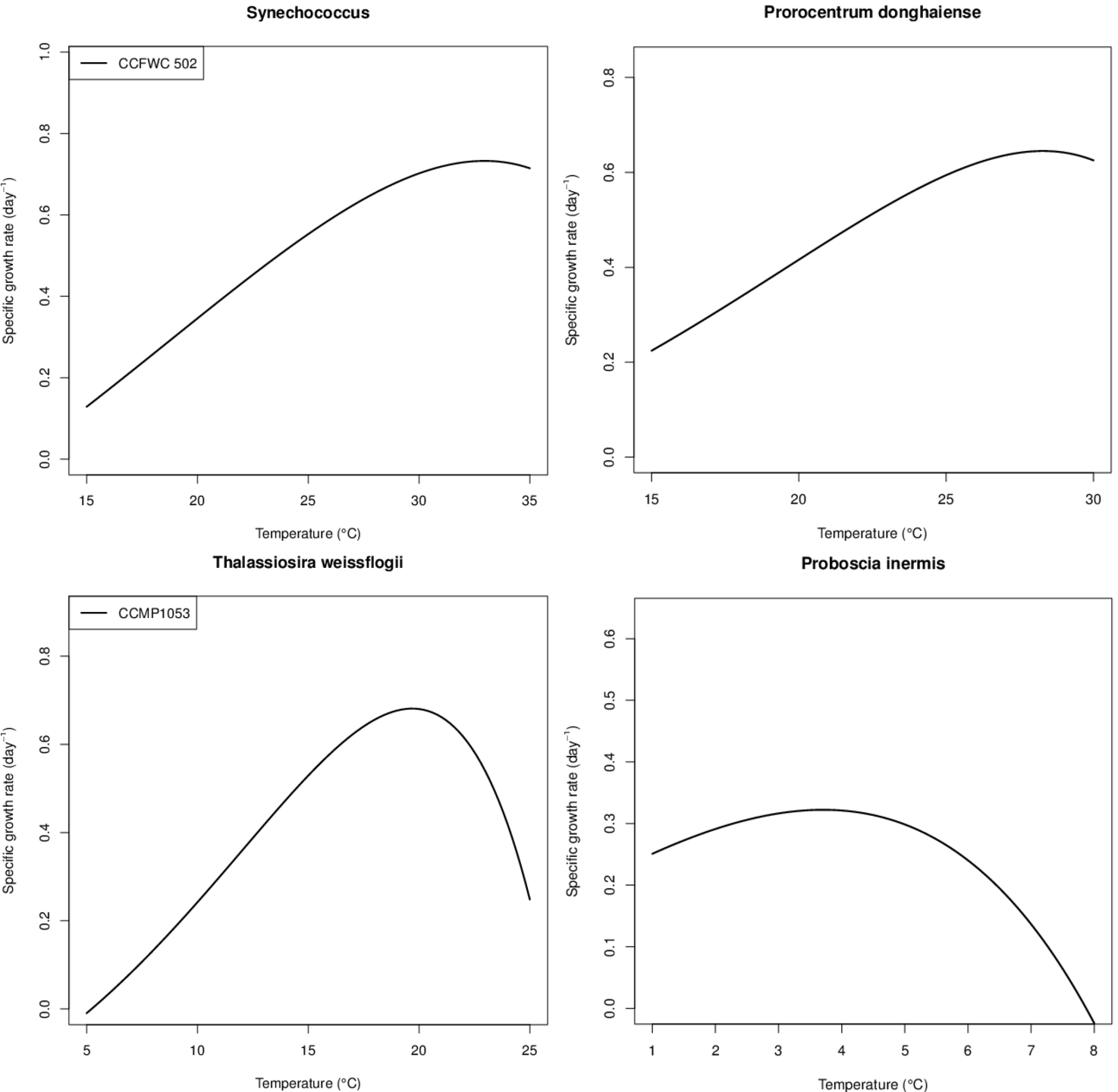

Supplement: Figure S2 — Intraspecific variation in thermal reaction norms for species in which only one strain was available. (TIF) [file pone.0063091.s002.tif]

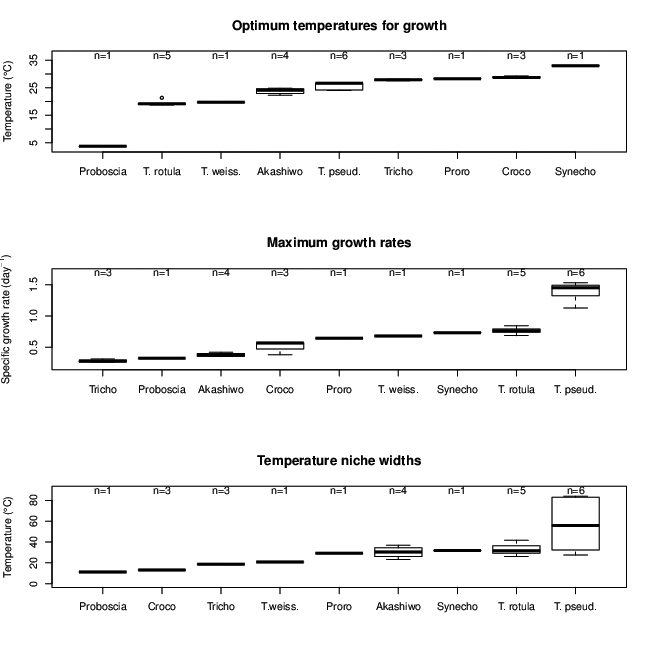

Supplement: Figure S3 — A summary of temperature optima, maximum growth rates and niche width – expressed as box and whiskers plots - for each of the species used in our study. The black bands denote the median value, the bottom and top of the box represent the 1st and 3rd quartile of the data, respectively. The ‘whiskers’ extending from the boxes indicate the positions of the lowest & highest values in the data. If the sample size is small enough, the whiskers may not appear (e.g. if there are only 3 equally spaced points, the value represented as the 1st quartile is the lowest value). (TIF) [file pone.0063091.s003.tif]

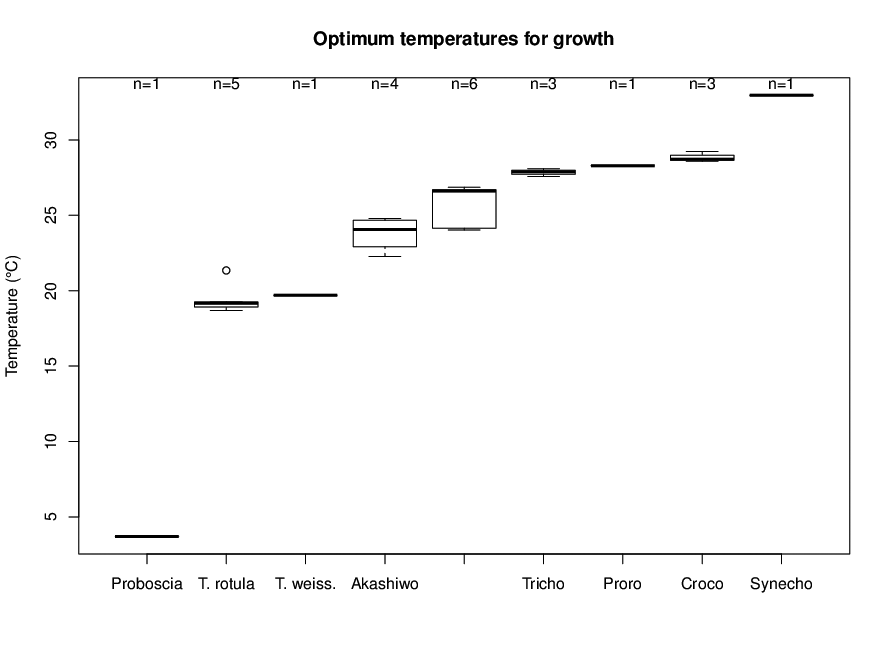

Supplement: Figure S4 — A summary of temperature optima (°C) obtained here (red boxes) and from the literature (blue boxes), expressed as box and whiskers plots, for each of the species used in our study (red). The thick black line in each box represents the median temperature. (TIF) [file pone.0063091.s004.tif]

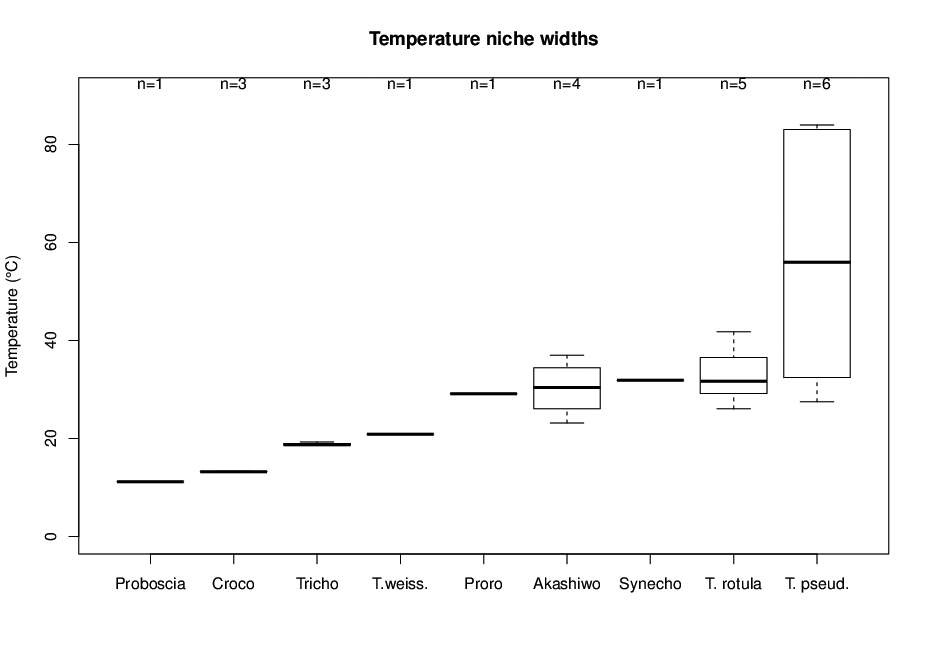

Supplement: Figure S5 — A summary of niche width (°C), expressed as box and whiskers plots - for each of the species used in our study. (TIF) [file pone.0063091.s005.tif]

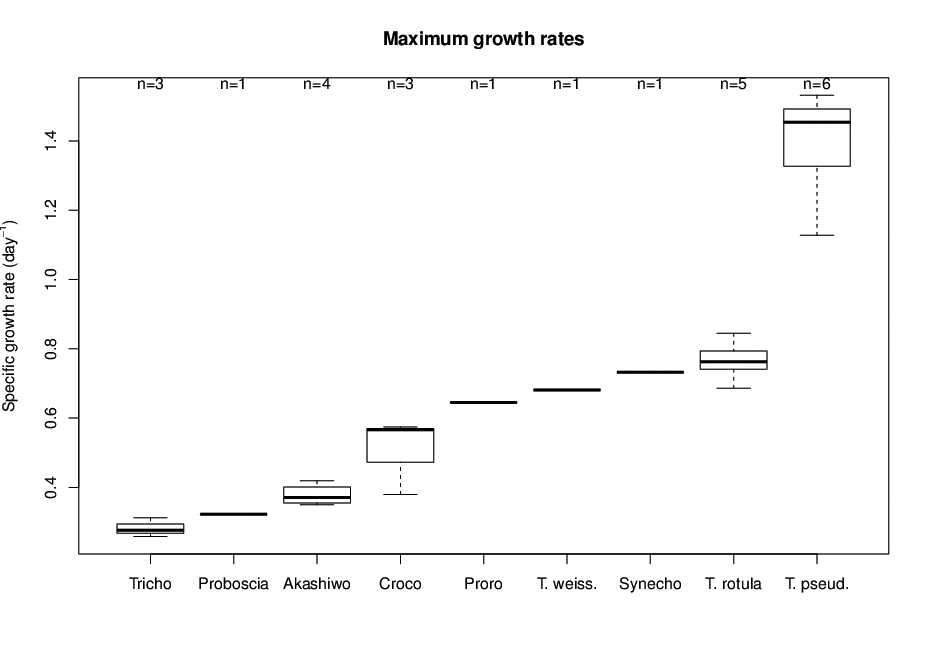

Supplement: Figure S6 — A summary of maximum growth rate expressed as box and whiskers plots - for each of the species used in our study. (TIF) [file pone.0063091.s006.tif]

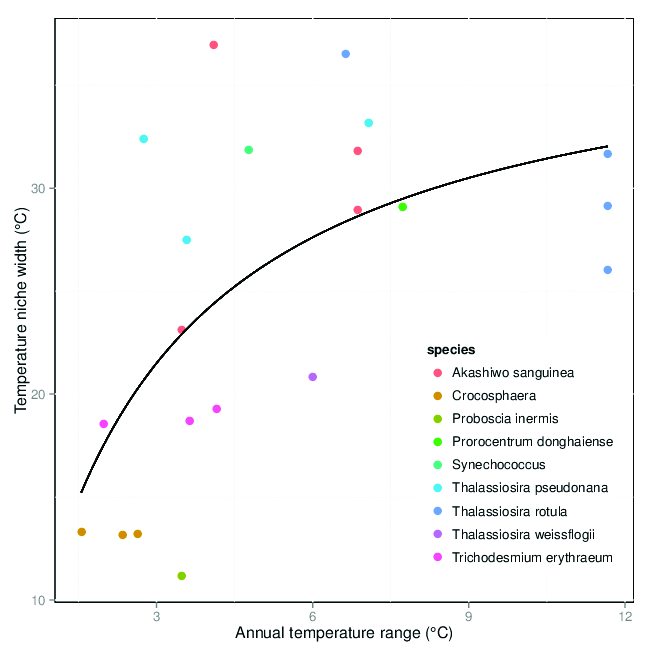

Supplement: Figure S7 — A plot of niche widths versus annual temperature range. Data points are coloured by species. This plot omits the niche widths that are poorly resolved (i.e. the 6 T. pseudonana +1 A. sanguinea strain). Niche widths increase as the annual temperature range increases, in accordance with the climate variability hypothesis. (TIF) [file pone.0063091.s007.tif]
