# Supplementary material for: Marine Phytoplankton Temperature versus Growth Responses from Polar to Tropical Waters – Outcome of a Scientific Community-Wide Study
Source: PLoS One. 2013 May 21;8(5):e63091. doi: 10.1371/journal.pone.0063091 (PMC3660375; doi:10.1371/journal.pone.0063091)
Supplement: Appendix S1 — (DOCX) [file pone.0063091.s008.docx]

*Appendix S1*

References of the prior lab culture studies used by Thomas et al. (2012) for their analysis of thermal traits from the literature.

Berges JA, Varela DE, Harrison PJ (2002) Effects of temperature on growth rate, cell composition and nitrogen metabolism in the marine diatom *Thalassiosira pseudonana* (Bacillariophyceae), Mar Ecol Prog Ser 225: 139-146.

Breitbarth EA, Oschlies A, LaRoche J (2007) Physiological constraints on the global distribution of *Trichodesmium* – effect of temperature on diazotrophy. Biogeo 4: 53-61.

Chappell PD, Webb EA, (2010) A molecular assessment of the iron stress response in the two phylogenetic clades of *Trichodesmium*, Environ Microbiol 12:, 13-27.

Falcón L, Pluvinage S, Carpenter EJ (2005) Growth kinetics of marine unicellular N_2_-fixing cyanobacterial isolates in continuous culture in relation to phosphorus and temperature, Mar Ecol Prog Ser 285: 3–9.

Ferguson RL, Collier A, Meeter D (1976) Growth response of *Thalassiosira pseudonana* Hasle and Heimdal clone 3H to illumination, temperature and nitrogen source, Chesapeake Sci 17: 148-158.

Guillard RRL, Ryther JH (1962) Studies of marine planktonic diatoms I. *Cyclotella nana* (Hustedt), and *Detonula confervacea* (Cleve) Gran, Can J Microbio 8: 229-239.

Krawiec RW (1982) Autecology and clonal variability of the marine centric diatom *Thalassiosira rotula* (Bacillariophyceae) in response to light, temperature and salinity, Mar Biol 69: 79-89.

Thomas WH, Dodson AN, Linden CA (1973) Optimum light and temperature requirements for *Gymnodinium splendens*, a larval fish food organism. Fish Bull 71: 599-601.

Thomas WH, Dodson AN (1974)Effect of interactions between temperature and nitrate supply on the cell division rates of two marine phytoflagellates, Mar Biol 24: 213-217.

Thompson PA, xin Guo M, Harrison PJ (1992) Effects of variation in temperature. I. On the biochemical composition of eight species of marine phytoplankton. J Phycol 28: 481-488.
